# Remembering James Barber (1940–2020)

**DOI:** 10.1007/s11120-022-00919-6

**Published:** 2022-05-09

**Authors:** Peter J. Nixon, Alison Telfer

**Affiliations:** Sir Ernst Chain Building – Wolfson Laboratories, Department of Life Sciences, Imperial College London, S. Kensington Campus, London, SW7 2AZ UK

**Keywords:** Photosynthesis, Photosystem II, Water oxidation, Mn cluster

## Abstract

James Barber, known to colleagues and friends as Jim, passed away in January 2020 after a long battle against cancer. During his long and distinguished career in photosynthesis research, Jim made many outstanding contributions with the pinnacle achieving his dream of determining the first detailed structure of the Mn cluster involved in photosynthetic water oxidation. Here, colleagues and friends remember Jim and reflect upon his scientific career and the impact he had on their lives and the scientific community.

**Supplementary Information:**

The online version contains supplementary material available at 10.1007/s11120-022-00919-6.

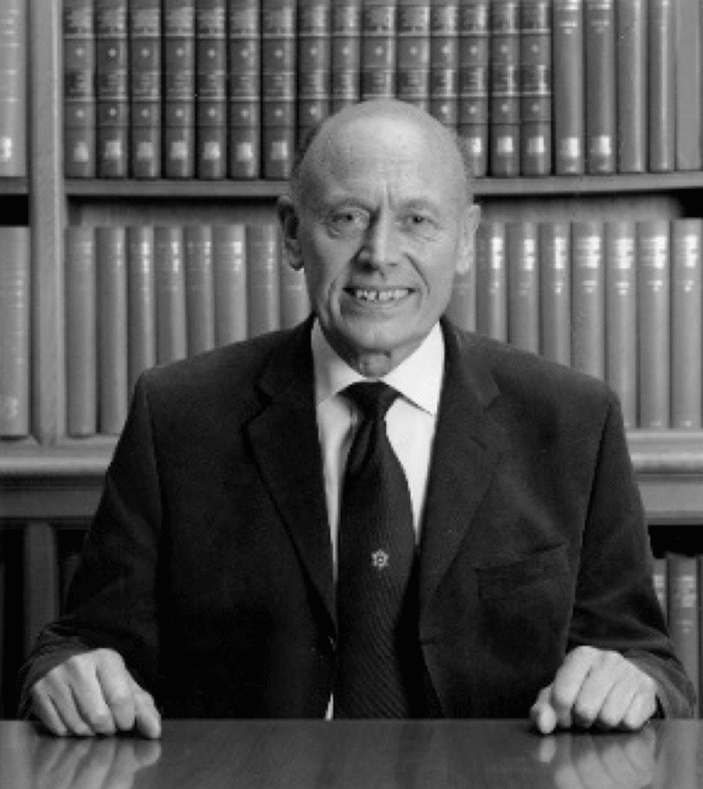

## Jim Barber’s scientific career

### Early years

**Fig. 1:**
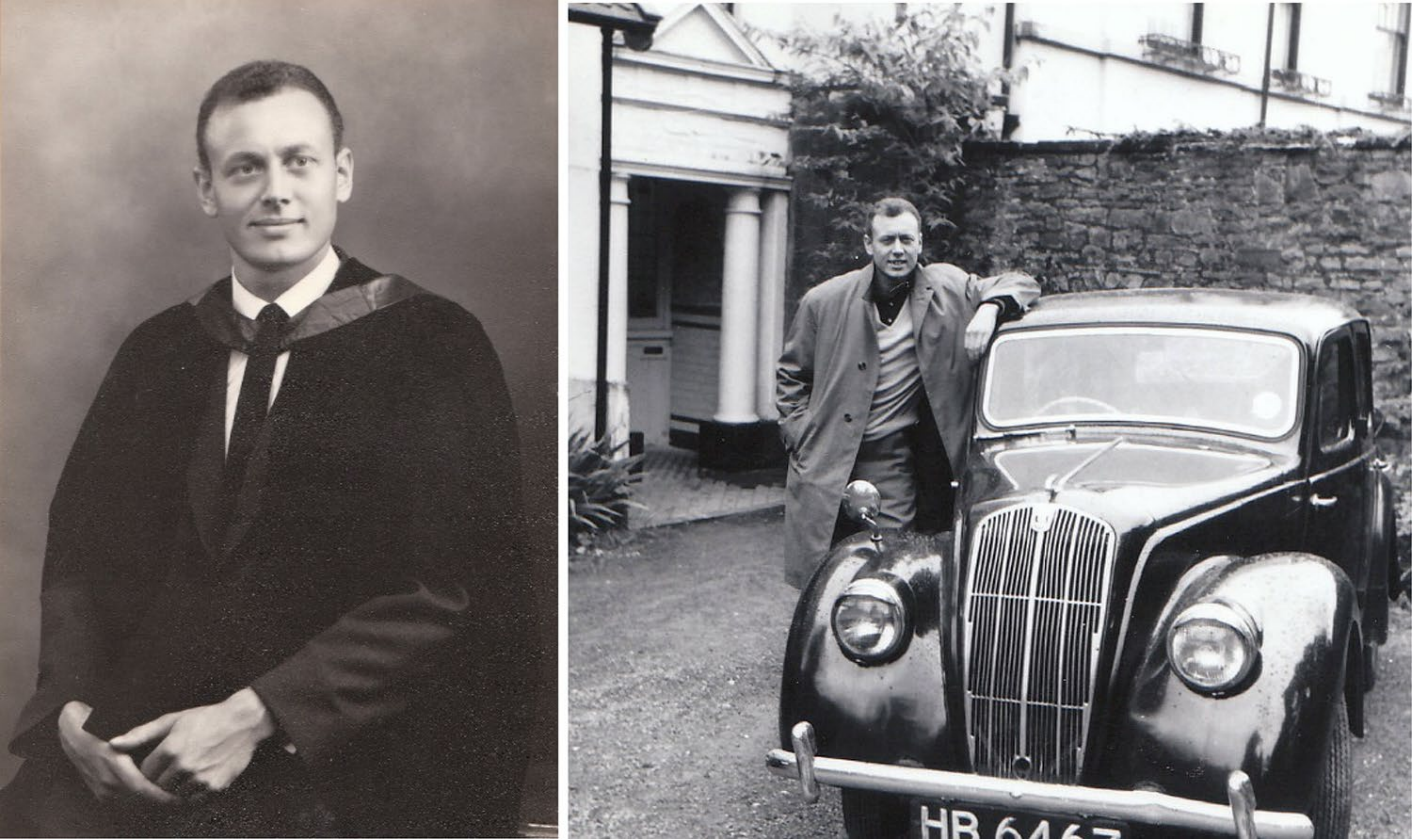
(left panel) Jim’s graduation photo, University College, Swansea; (right panel) Jim at Swansea with his 1948 Morris Series E

Jim was born on July 16th 1940 and grew up in modest circumstances in the town of Portsmouth on the south coast of England. His route into academic life was far from conventional. He left school at 16 to take up an engineering apprenticeship at the Signals Research and Development Establishment of the Ministry of Defence. Then, following evening classes at the local technical college, Jim was awarded a Technical State Scholarship to study chemical engineering at University College, Swansea. After converting to pure chemistry, Jim graduated with a BSc degree in 1963 (Fig. 1). Helped by the award of a scholarship from the Nuffield foundation, Jim converted to the biological sciences and moved to the University of East Anglia in Norwich where he obtained a Masters degree in biophysics followed by a PhD degree. Jim’s PhD supervisor, Jack Dainty, a nuclear physicist who had likewise converted to the biological sciences, encouraged Jim to work on the light reactions of photosynthesis, in part because of the recent Nobel Prize awarded to Calvin in 1961 for his work on the dark reactions. Suitably inspired, Jim’s first project in the area was to investigate the role of photosynthesis in the active transport of ions into the green alga *Chlorella pyrenoidosa*, with his very first paper published in Nature (Barber 1968). As Jim explained later, the experimental work required the insertion of a fine glass electrode into an algal cell to measure the membrane potential. This proved to be a somewhat challenging task and so Jim was indebted to the tenacity of Lyn, his future wife, for having the patience to carry this out.

Following his PhD, Jim was awarded a Unilever European Fellowship of the Biochemical Society to work at the State University of Leiden in the lab of Professor L. M. N. Duysens, a leading biophysicist in photosynthesis research. Here, Jim discovered the phenomenon of salt-induced delayed light emission from chloroplasts which provided early evidence for a role for the membrane potential in controlling charge recombination reactions in photosystem II (PSII) (Barber and Kraan 1970).

### Imperial College

**Fig. 2:**
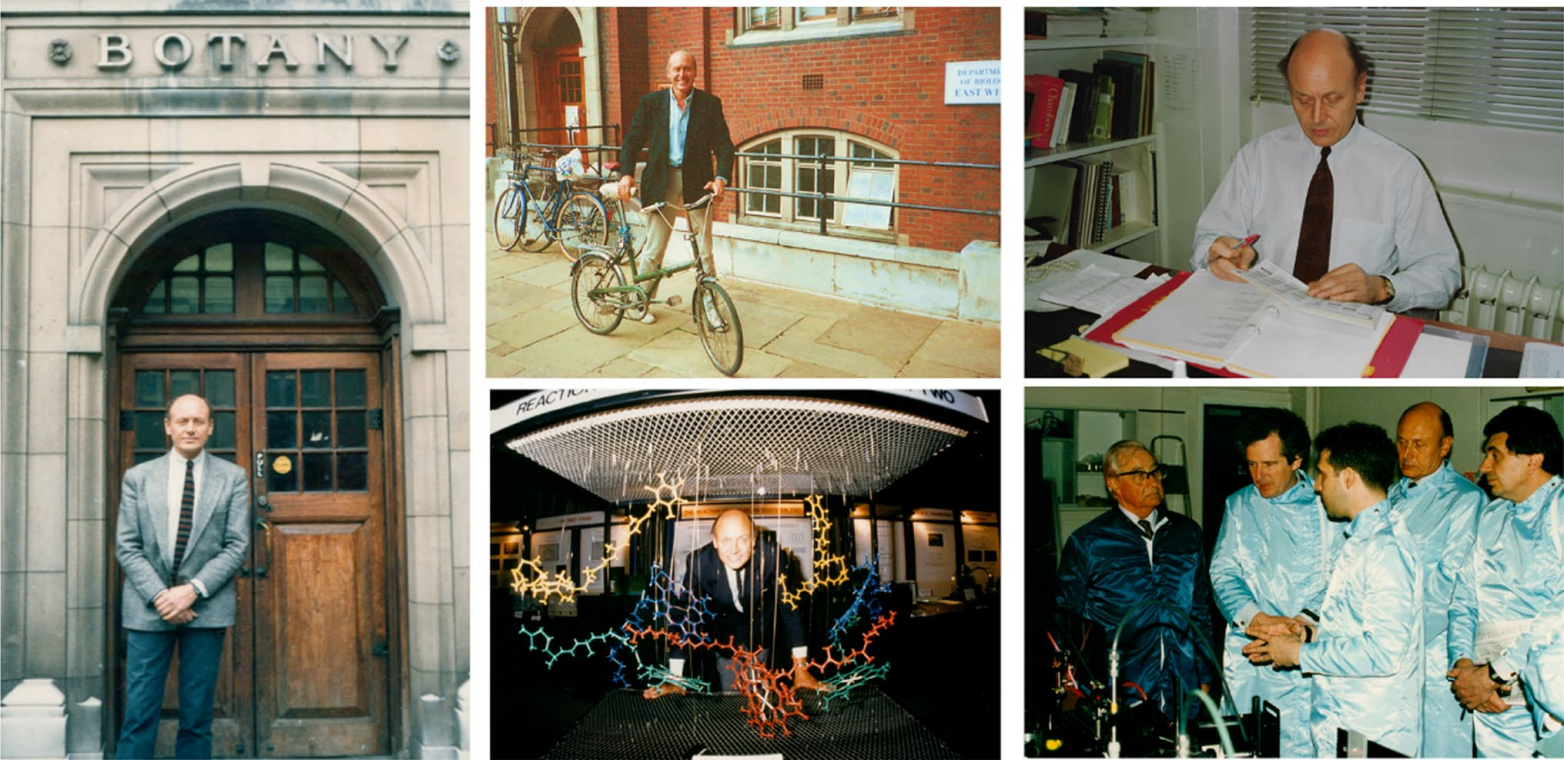
Clockwise from the left panel: Jim outside the entrance to the former Department of Botany at Imperial College; arriving for work; in his office; with Lord Porter (on left) and David Klug (third from left) during a visit by the cabinet minister, William Waldegrave, (second from left) to the laser lab in the Centre for Photomolecular Sciences and standing behind a wire model of the cofactors of the PSII reaction center at an evening soirée at the Royal Society in the summer of 1989

In 1968 Jim was appointed Lecturer in the then Botany Department at Imperial College in London (Fig. 2) where he continued his studies on the effect of ion gradients on prompt and delayed fluorescence from chloroplasts. Although Imperial provided an intellectually stimulating environment, there were limited facilities within the Department to perform the experimental work. Indeed, Jim’s first dark room was literally a small broom cupboard where the cleaners kept their mops and buckets, and it took Jim a year before he could afford to buy a pH meter. Jim’s engineering skills also came in useful for building his own stop-flow system and phosphoroscope for measuring chlorophyll fluorescence.

By the late 1970s Jim had established a vibrant research group. Their studies led to several seminal papers highlighting the importance of surface charge in controlling the stacking of thylakoid membranes (Barber and Chow 1979) and the interaction of PSI and PSII (Barber 1980a). Theoretical considerations of the electrostatic interactions between protein complexes led Jim to propose an elegant molecular mechanism for how protein phosphorylation could control state transitions, a regulatory process that balances the excitation of PSI and PSII (Barber 1982). Jim’s early work on the role of surface charge in regulating membrane dynamics is still highly influential today (Puthiyaveetil et al. 2017).

In the early 1980s, with funding from the then UK Agricultural Research Council (ARC), Jim and his group began to study fundamental aspects of the thylakoid membrane, especially the lipid composition, its fluidity and the impact of low temperature on the diffusional properties of the membrane and photosynthetic efficiency (Barber 1983), with the overall aim to understand the factors that allowed plants to grow at lower temperatures.

**Fig. 3:**
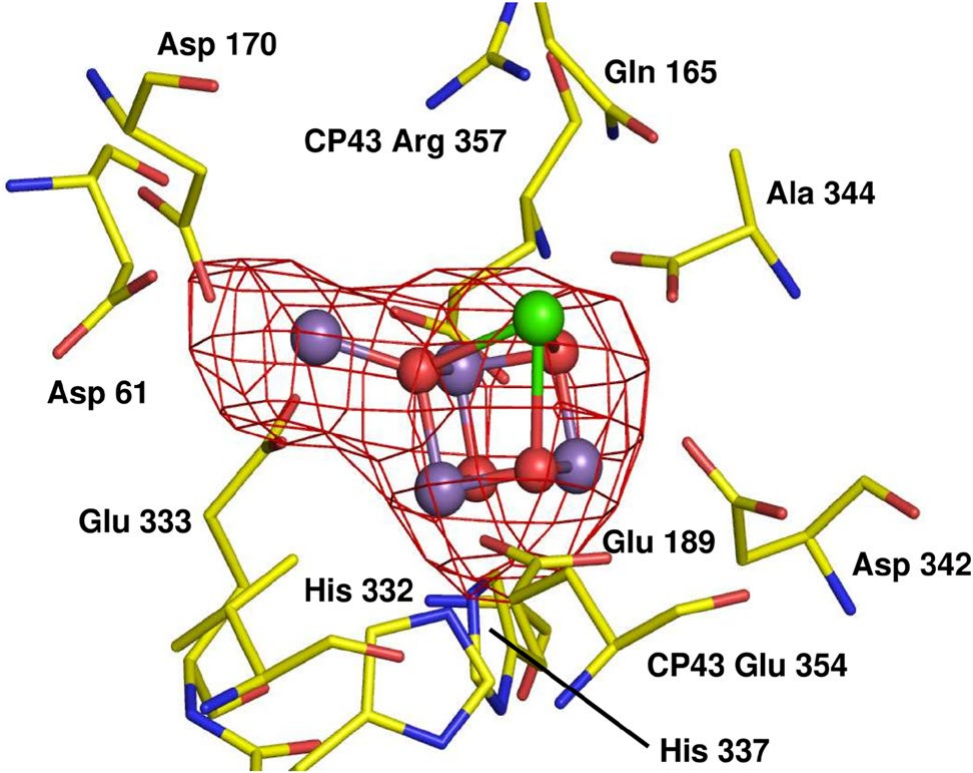
The original model of the Mn cluster from Jim’s 2004 paper (Ferreira et al. 2004; PDB 1S5L), drawn with the Mn edge anomalous difference Fourier map, showing density corresponding to the four Mn atoms (in purple), the oxo bridges (in red) and the single calcium ion (in green). Residues within D1 and CP43 in the vicinity of the cluster are indicated

By the mid-1980s, Jim changed his focus from lipids to understanding the properties of the oxygen-evolving PSII complex, which with typical panache he termed ‘The Engine of Life’ (Barber 2004). Jim set himself the ambitious goal of elucidating the structure and mechanism of PSII. Jim knew that climbing this particular scientific mountain would require a multidisciplinary approach, involving the biochemical isolation and characterization of PSII complexes, spectroscopic studies (largely done through a longstanding collaboration with the Centre for Photomolecular Sciences at Imperial College established by Lord George Porter, Nobel Laureate), the analysis of PSII mutants and, ultimately, the elucidation of protein structures (with the help of So Iwata, Marin van Heel, Werner Kühlbrandt and others). All these efforts culminated in 2004 with the publication in *Science* of a structural model for cyanobacterial PSII at a resolution of 3.5 Å obtained in collaboration with So Iwata who was now at Imperial College (Ferreira et al. 2004). This was a landmark achievement as it provided the first glimpse of the manganese cluster that catalyzes water oxidation. Somewhat surprisingly, the cluster was composed of an unprecedented Mn_3_Ca cubane-like structure attached to a more distant ‘dangler’ Mn through oxo bridges (Fig. 3). Although initially met with some scepticism, the key aspects of the model have now been confirmed (Umena et al. 2011) and great strides have continued to be made to improve the structural resolution to a level that now allows insights into the mechanism of water oxidation at the atomic level (Suga et al. 2019).

Although determining the structure of PSII became Jim’s major research focus, Jim’s energy and curiosity led to important contributions in other areas. These included early time-resolved measurements of energy transfer in photosynthesis in collaboration with Lord Porter (Beddard et al. 1975), developing remote sensing methods for mineral exploitation (Beauford et al. 1975) and for probing plant productivity (Horler et al. 1983), investigating the mechanisms of photoinhibitory damage to PSII (Barber and Andersson 1992) and determining the structures of cyanobacterial PSI and PSII supercomplexes with attached light-harvesting antennae (Bibby et al. 2001, 2003a, b). A full description of Jim’s incredibly productive research career can be found in Jim’s book ‘The Big Bang of Evolution and the Engine of Life: Climbing a Mountain’, published just before his death (Barber 2020).

### Italy and Singapore

Jim never retired. He just found new challenges. Towards the end of his research career at Imperial College, Jim was appointed Visiting Professor at the Politecnico di Torino and with the help of his postdoc at the time, Cristina Pagliano, established the BioSolar Laboratory. Here Jim and his colleagues began to isolate PSII-LHCII supercomplexes from plant chloroplasts for structural studies (Barera et al. 2012), work that is continuing to reveal important new insights (Albanese et al. 2017).

In 2008, Jim was appointed Visiting Professor to the School of Material Science and Engineering at Nanyang Technological University (NTU) where he helped establish the Solar Fuels Lab (SFL) with the aim to construct a robust, long-life technology to split water using solar energy (Gurudayal et al. 2015). It was a wonderful experience for Jim, now in the twilight of his career, as it allowed him to apply the basic principles of natural photosynthesis to artificial systems. Jim was a great advocate of developing an ‘artificial leaf’ that could capture solar energy to make a fuel at a scale that could replace fossil fuels (Barber 2009). His upbeat message was ‘if plants can do it, we can do it: it is only chemistry’. He was especially grateful to Bertil Andersson (President of NTU) and Freddy Boey (then Chair of School, but later Provost of NTU) for their support and always looked forward to traveling to NTU even when in poor health to work with the team of outstanding young scientists based there.

### A leader of the photosynthesis community

**Fig. 4:**
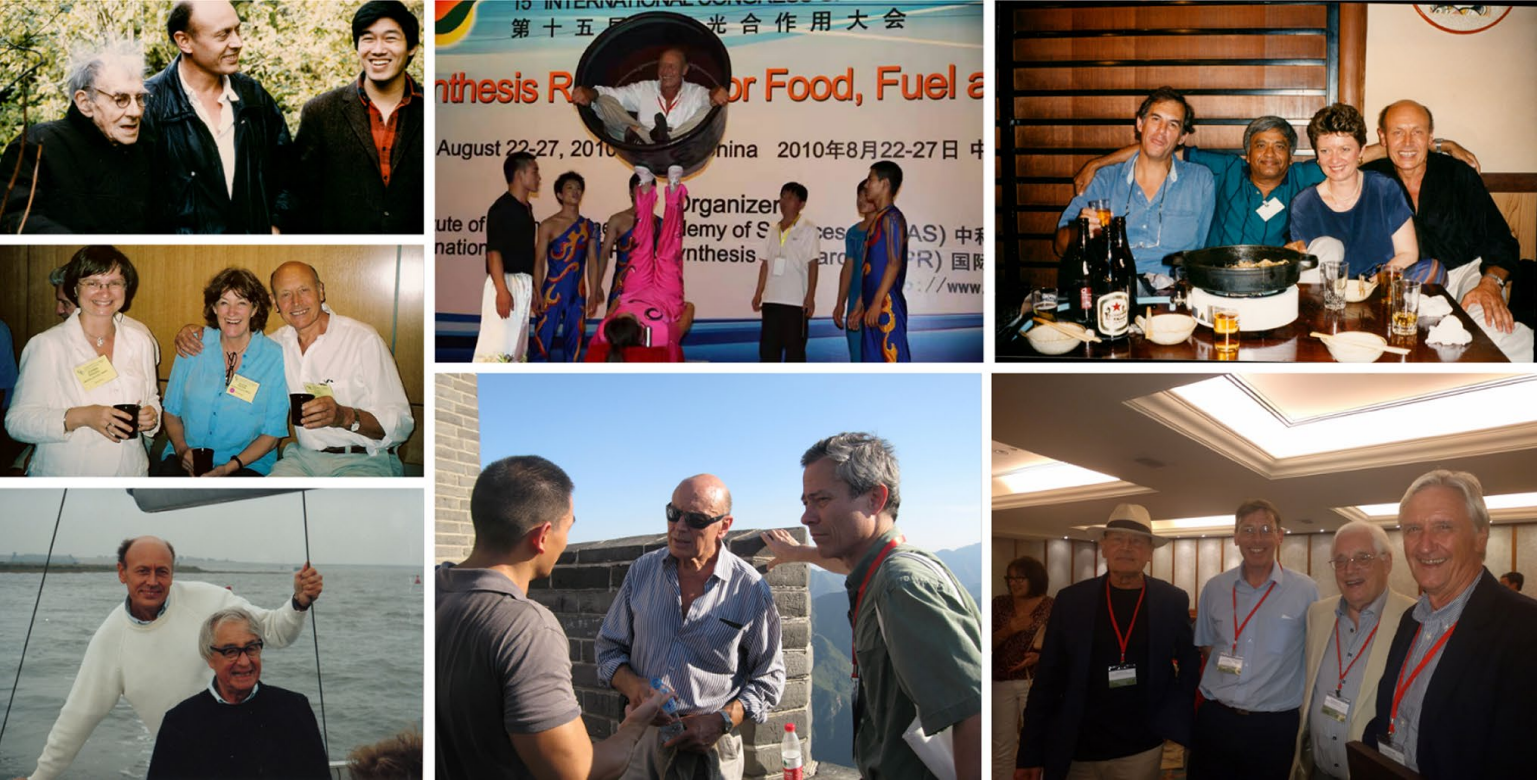
Jim with colleagues and friends. Clockwise from top left-hand corner: with Robin Hill (on left) and Eric Lam (on right) in 1986; in a barrel at the banquet of the 2010 International Photosynthesis Congress in Beijing, China (photo kindly provided by Wenqiang Yang); dinner with Tony Crofts, G. Govindjee (in center), and Christa Critchley after the 1992 International Photosynthesis Congress held in Nagoya, Japan; with (from left to right) Peter Nixon, John Walker and Les Dutton in Singapore 2016 (Lyn Barber can be seen in the background); with Gary Brudvig (on right) and a colleague on the Great Wall of China in 2010; sailing with Lord Porter and, finally, at the 2006 Photosynthesis Gordon Research Conference held at Bryant University, USA, with, left to right, Joanna Kargul and Alison Telfer

Jim was an extremely well-known, influential and highly respected figure in Photosynthesis Research. He served as President of the International Society of Photosynthesis Research from 2007 to 2010 and during his long career travelled widely, with his sabbatical stays at the Weizmann Institute (1980, 1983), University of Rosario, Argentina (1982), Berkeley (1988) and lecture tours to India, China and the Soviet Union enabling him to forge deep friendships with scientists from all parts of the world (Fig. 4). Jim was always keen to support young scientists from across the globe and he and Lyn were always welcoming hosts to the large number of visitors who spent time in his lab.

**Fig. 5:**
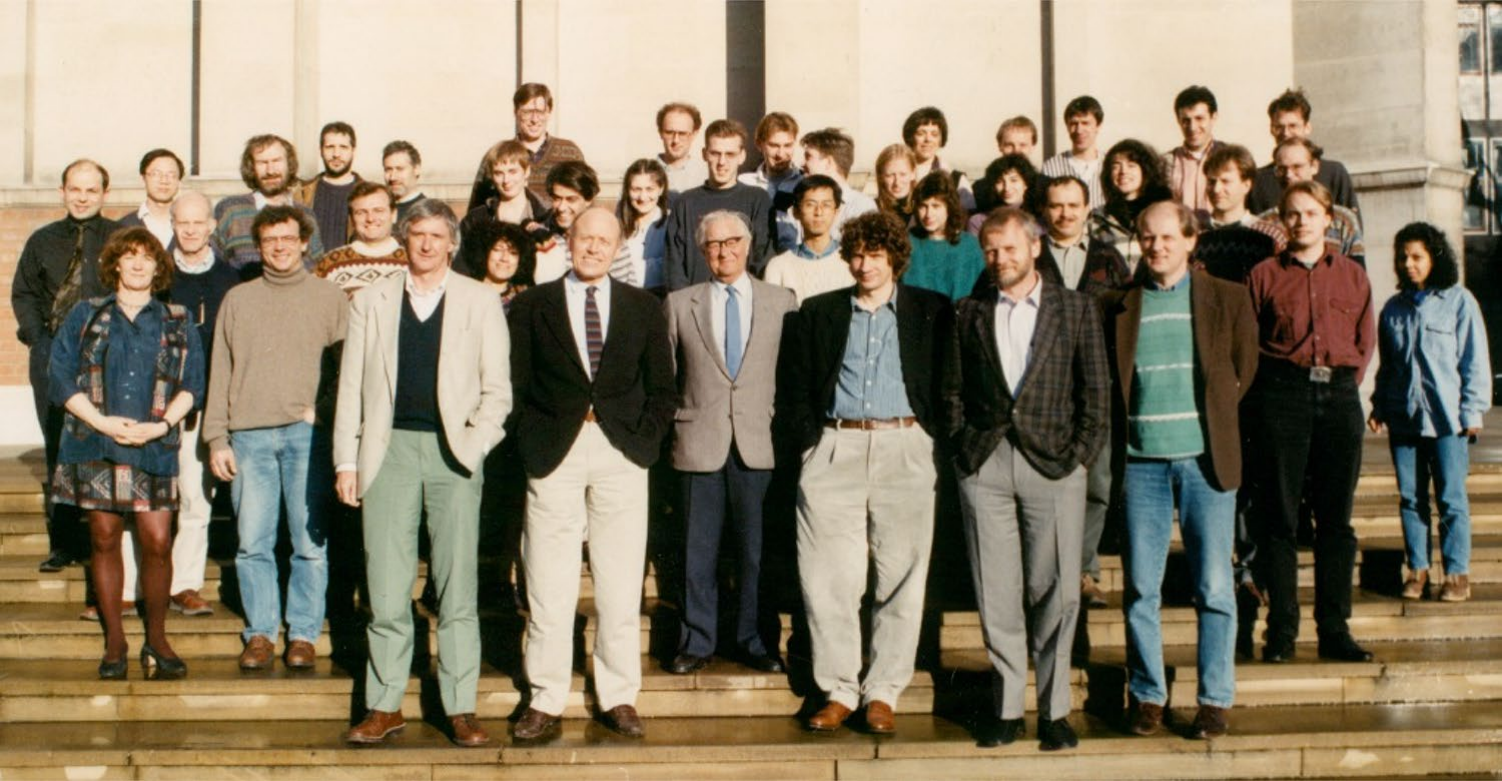
Discussion meeting involving Visiting Professors to the Department of Biochemistry, March 1995. On the front row from left to right: Les Dutton, Jim Barber, Lord Porter, Werner Kühlbrandt, Hartmut Michel and Bertil Andersson

Jim was an energetic promoter and supporter of photosynthesis research. He was editor of the well-known book series ‘Topics in Photosynthesis’ covering 10 volumes which ran from 1976 to 1988 and he organized many scientific meetings which are the lifeblood of the scientific community. He was especially proud of the AFRC Robin Hill Photosynthesis Meetings held at Imperial College in the 1990s, which helped promote photosynthesis research in the UK. After Jim became Head of the then Biochemistry Department at Imperial in 1989 (now part of the Department of Life Sciences) he also organized a discussion meeting each year for Visiting Professors (Fig. 5). This eventually evolved with the encouragement of Les Dutton into the annual ‘Bunty Plot’ meeting with the focus on bioenergetics and an emphasis on informality and discussion. More recently, Jim organized five International Workshops on Solar Energy for Sustainability held at the Institute of Advanced Studies at NTU in Singapore.

### Prizes and achievements

Jim was a prolific researcher and wrote more than 600 papers, with a particular favourite a review he wrote with his son Neil, a surgeon, on the potential benefits of dietary lycopene for preventing prostate cancer (Barber and Barber 2002).

**Fig. 6:**
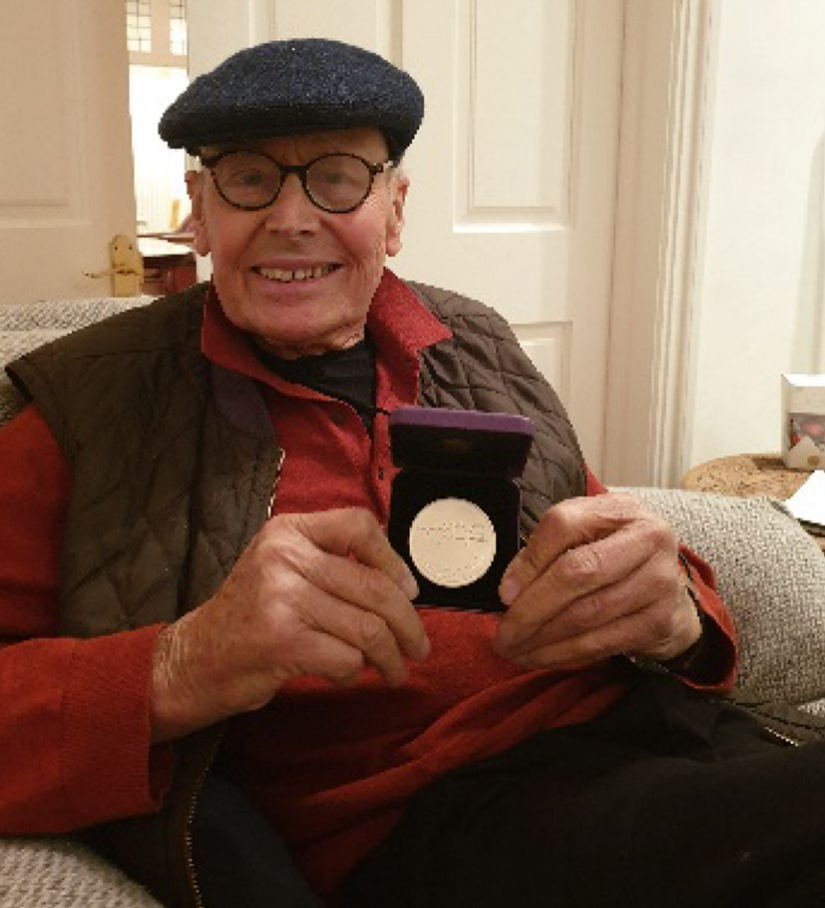
Jim in November 2019 proudly showing the UK Biochemical Society Heatley Medal

Jim’s considerable scientific achievements were recognized by the award of several prizes including the Flintoff Medal of the Royal Society of Chemistry (2002), the ENI award for Energy and the Environment (2005), the Biochemical Society Novartis Medal and Prize (2006), the Wheland Medal and Prize from the University of Chicago (2007), the Royal Society of Chemistry Interdisciplinary Medal and Prize (2013), the Porter Medal (2016), the Communication Award of the International Society of Photosynthesis Research (2016) and the UK Biochemical Society Heatley Medal and Prize (2020) (Fig. 6). In addition, Jim was elected a Fellow of the Royal Society of Chemistry (FRSC) in 1980, a Member of the Academica Europaea (MAE) in 1989, Selby Fellow of the of the Australian Academy of Science in 1995, Foreign Member of the Royal Swedish Academy of Sciences in 2003 and, to his great delight, a Fellow of the Royal Society (FRS) in 2005. Jim also received Honorary Doctorates from the University of Stockholm (1992), University of East Anglia (2010) and Nanyang Technological University, Singapore (2017).

Jim recognized that all these honours would have been impossible without the help and input of the many PhD students, research fellows, collaborators and support staff who worked in his group and the academic visitors who came to his lab. Jim was especially proud that many of his 50 former PhD students and research fellows went on to become successful research scientists in their own right and that the Photosynthesis Research Lab which he established on the 7th floor of the Sir Ernst Chain Building over 30 years ago when he became Head of Department and Ernst Chain Professor of Biochemistry continues to flourish with Bill Rutherford, Peter Nixon, Jasper van Thor, James Murray and Tanai Cardona all working on various aspects of photosynthesis.

### Jim away from the lab

**Fig. 7:**
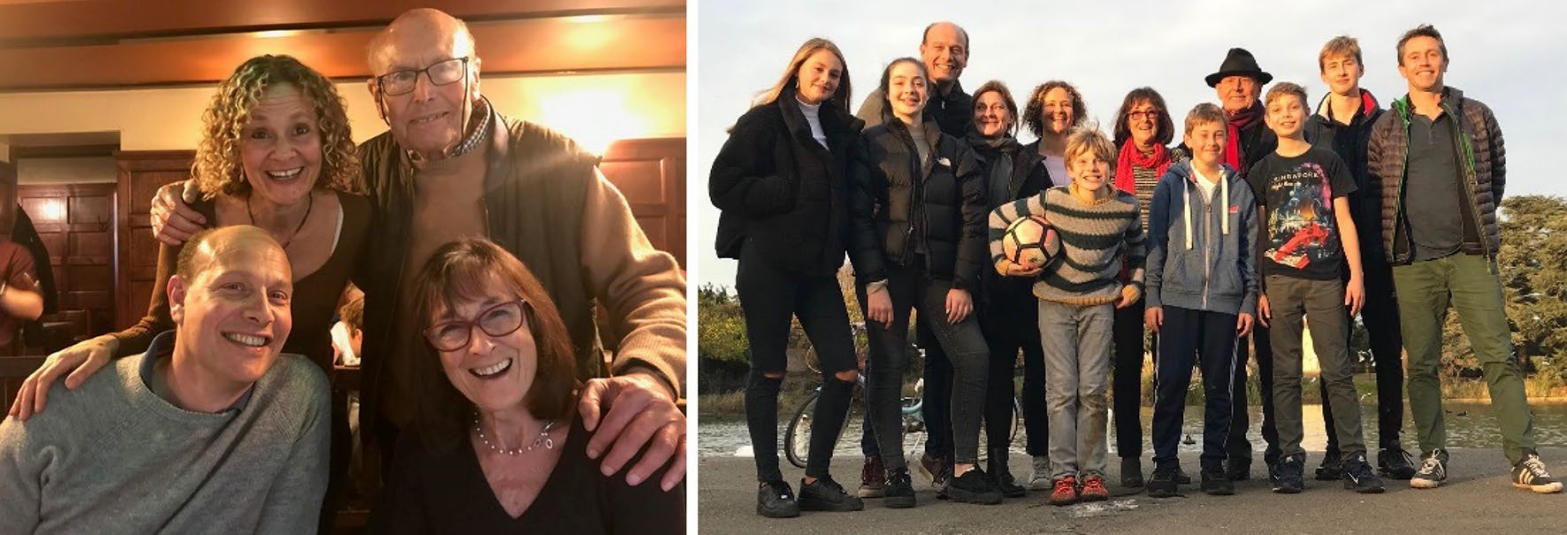
(left panel) Jim and Lyn with Neil and Julie in 2019; (right panel) Jim, Lyn and family at Gunnersbury Park, London in 2018

Jim’s incredible energy, his enthusiasm for new challenges and capacity for hard work extended well beyond the lab. Jim played an active role as Head of the Biochemistry Department in modernizing the Department and establishing the Centre for Structural Biology. Away from work, Jim and Lyn renovated a cottage in rural Sussex that became a special place for them and their family. Here, Jim and Lyn hosted lab retreats, held birthday parties and enjoyed entertaining friends. But above all, Jim and Lyn loved spending time with their family: daughter Julie and her husband Tom; son Neil and his wife Lizzie; and grandchildren Zoe, Jessica, Ben, Toby, Harry and Theo (Fig. 7). Photosynthesis has lost one of its great protagonists, but Jim led an extremely well-lived life and leaves behind many legacies and fond memories.

### Reminiscences

*Dick Malkin (University of California, Berkeley, USA)* I first met Jim at a Gordon Conference in 1971. Little did I know at that time that this was the beginning of a friendship that would extend 50 years and would result in Jim being my closest friend.

**Fig. 8:**
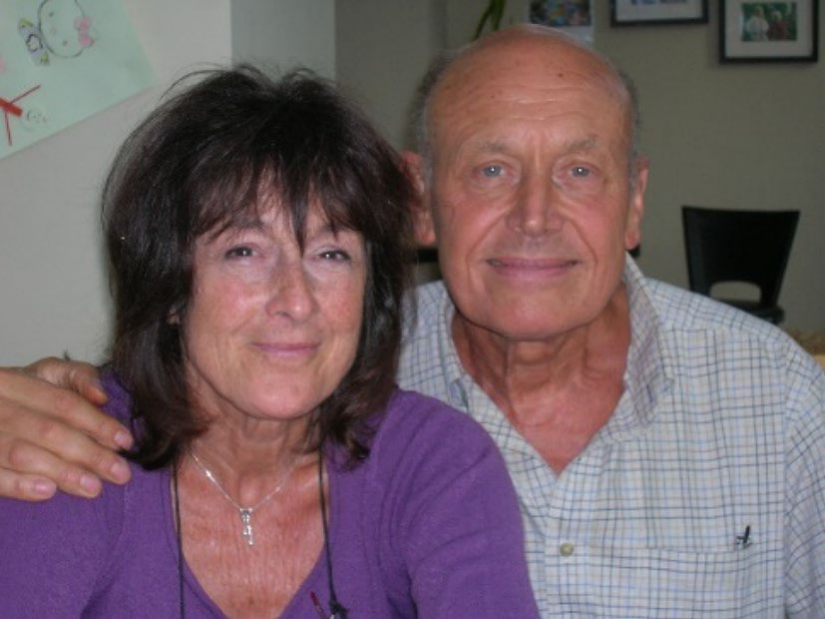
Jim and Lyn

Over our long friendship, my wife Carole and I managed to see Jim and Lyn regularly in London (Fig. 8). Our favorite time was the winter because of the active cultural scene since Carole loved the theaters and I loved opera. When we first started visiting, Lyn and Jim were living in a large Victorian house on Woodstock Road in Chiswick, a close-in suburb of London. The house was amazing because earlier it had been converted into a large number of flats, but Jim was determined to convert it back into a home with a large number of bedrooms. I think the number was between six and eight. It was during this renovation project that I learned Jim was a master handyman with the skills of a carpenter, an electrician, a plumber, a tiler, and on and on. The house also had a small garden in the back where Jim tended a small number of crops, growing fruits and vegetables. My best memory of this time is seeing Jim in his bathrobe in the morning going out to pick fresh rhubarb and cooking it for breakfast.

Jim later moved from Woodstock Road to Dukes Avenue and then to St. Albans Avenue in Chiswick and also purchased property in Sussex that had a cottage on it. The cottage became a high point for the family. The seventeenth–eighteenth century cottage brought out all of Jim’s building skills as he modernized it with new bathrooms and bedrooms. And there was a large vegetable garden that became the love of his life, yielding veggies for the year. Jim’s potatoes were better than anything in local markets.

An aside- Jim came to California in the early 1980s to attend a winter Gordon Conference. He came to Berkeley to give a seminar on the same trip and we planned to drive to Santa Barbara. Dave Knaff was also with us. There was one problem—the Oakland Marathon was on Sunday morning and I was running in it. I also told Jim there was a half-marathon (13 ½ miles) as part of the race and Carole and Dave were going to run in that race. When I told Jim, he immediately said he would run with them. I asked Jim what was the longest he had ever run and he said “4 miles,” which probably meant 2–3 miles, but he insisted he could do it. On Sunday morning we all got up at 5 am to go. Another problem came up—it was pouring outside with heavy winds. I said I was still going and Jim looked around for some support for staying home but the others said they were also going. After that, he knew he would be joining us. Much later in the morning, we all finished and were pretty tired, except for Jim, who looked much better than any of us. He explained he would find someone in the race to talk to and after a while move on to someone else and before he knew it, he was done. Jim loved talking to people and was the best storyteller I ever knew. I also knew he would never quit because he would never give up on anything.

During the 1980s, Jim changed his research direction to studies of photosystem II. He threw all of his energies into this massive project with the goal of obtaining a complete structure for this complex. This work, done in collaboration with So Iwata, culminated in the mid-1990s with a complete X-ray structure of the photosystem with the first elucidation of the structure of that manganese oxygen-evolving complex, the so-called Holy Grail of Photosynthesis.

In the summer of 2012, all this dramatically changed when Jim was diagnosed with colon cancer, stage 3. This was serious, and he was told he might have only 6 months to 1 year to live. During this time, Carole and I talked with Jim often, and Jim saw Carole as a role model. Carole had breast cancer in the 1980s and was a survivor. Jim would say “If Carole could beat cancer, I will too.” He went through a series of treatments that went on for years, including surgery, chemotherapy, and radiation, but on the whole, they were not working. Jim said, “I am not giving up,” and he continued to work. This included writing papers, organizing meetings, and traveling to Singapore where he was a Visiting Professor. He said he loved his science and would not give it up.

Carole was diagnosed with melanoma in 2012 but it was stage 1 with no metastasis. However, in 2014, the cancer returned in stage 4 and she was told there were no treatments. Carole passed away on September 7, 2014. I called Jim and Lyn, knowing it would be as hard on Jim as it was on me.

Over the next several years, I made trips to London, trying to keep Jim’s spirits up. We would have long talks during his chemotherapy sessions on the mechanism of oxygen evolution, which became his prime focus now that the structure of the oxygen complex was known. He really loved the field of photosynthesis and had started on artificial photosynthesis, hoping to relate his results to the problem of global warming and climate change. He was frustrated by the unwillingness of scientists and politicians to deal in any practical way with what he considered to be the major problem of the world.

Jim died on January 5, 2020 at home with his family around him. When Lyn called me to tell me he had passed away, I felt an overwhelming loss. He had told me on one of my last visits that he looked on me as the brother he had never had, and I knew we were much more than friends. Jim wanted very much to live to 80 so he could have another birthday party, but the situation was not good and he could not cross the finish line.

*Alison Telfer (Imperial College London, UK)* My first encounter with Jim was an amazing photobiology meeting held at Imperial College in 1969. All the famous European scientists of the photosynthesis world were there: Pierre Joliot, Horst Witt, Mordechai Avron to name but a few. How did Jim do this? He wasn’t even specializing in photosynthetic electron transport or water-splitting at the time and it was only his second year as a lecturer at Imperial College. I don’t think I even spoke to him that day. It was 3 years later when I was looking for a postdoc position in London that I found an advert for someone to finish a postdoc grant, as the current appointment, Peter Gregory, had decided to leave the research field. Peter had moved to Imperial after his PhD at Kings College, where I was a postdoc, and he used to ring me up for advice on how to isolate chloroplasts, etc. So, I cheekily thought ‘Well I have been doing that job anyway for the last year’ so I went for the interview and took over the post. Lack of academic positions in the UK during the 1970s meant I ended up staying with Jim as a research assistant for the rest of my working life. Jim was very flexible about working hours and surprisingly Imperial had a very good day nursery, which meant I could continue my research career whilst raising a family.

The setting up of the photobiology meeting in 1969 was an example of Jim’s flair for getting scientists together. He invited giants of the field to visit the lab in Beit Quad overlooking the Royal Albert Hall while we were still in the old Botany Department. I remember both Paul Mathis and Philip Thornber writing on a board held up by clamps in our old-fashioned lab as we perched on lab stools. Also, I will never forget the lecture at Imperial by Harmut Michel on the first X-ray structure of a photochemical reaction centre. Jim encouraged inter-College research discussions within London University and then there were all the other meetings he ran: D1/D2 reaction centre meetings, AFRC Photoinhibition meetings, Photochemical and Photobiological Research Group meetings, and a NATO workshop at Spetses.

The early interaction with Sir George Porter at the Royal Institution during the 1970s led to Lord Porter bringing his Laser Group to Imperial after he became the President of the Royal Society (Fig. 2). I believe the old Imperial College Botany Department must have been the only one in the world with a picosecond laser set up studying charge separation in isolated photosynthetic reaction center complexes.

Our move in 1989 to the Biochemistry Dept at Imperial College led to greater expansion of the meetings Jim organized—culminating in the annual Bunty Plots—which he kept going throughout his illness—and which brought together the greatest thinkers in the world of photosynthesis and bioenergetics research. More recently, Jim became a great exponent of Artificial Photosynthesis and drew attention to the ongoing problems of climate change and need for clean energy (Barber 2009).

Jim and I worked together from 1972 until he and I retired from the lab at about the same time. His kindness was much appreciated when he organised a meeting celebrating my 65th birthday and my work at Imperial by inviting many of the colleagues I had worked with in Europe and further afield. I helped organize his 75th birthday party in 2015—I managed to get more than a 100 of his PhD students, postdocs, friends and colleagues and collaborators to come to the scientific meeting and the evening dinner. At the end of the scientific presentations Jim gave an impromptu speech on both his personal and working life which is available online (Online resource 1). Sadly, he didn’t make it to his 80th birthday though we had things in preparation for it. I think he realized he wouldn’t make it to his birthday so he organized another birthday meeting for me – my 75th—with scientific presentations and a party in the summer of 2019. It was a marvellous way to celebrate my scientific career and my 47 years of working with Jim.

*David Horler (Ottawa, Canada)* I remember vividly my first brief meeting with Jim when I applied for a PhD studentship. I was struck by his energy, enthusiasm and direct manner. I immediately felt the magnetism of the great man. In the 6 years I spent in Jim’s lab, he became my mentor and friend. Through the ensuing decades, despite long periods when we did not meet, we were always able to pick up where we left off as if the gap did not matter, and the magic never left. I worked in the remote sensing side of the lab and was proud to interact with Jim’s world class photosynthesis research group. To Jim, it was just a matter of scale, molecular or planetary, a perspective that surfaced again later when Jim’s focus turned to solar fuels and energy sources inspired by photosynthesis. Jim’s achievements and incredible drive, even during his last years when battling illness, were inspiring. Jim’s was truly a life well-lived, filled with purpose and capped by a towering legacy in science and in his wonderful family.

*Tony Larkum (University of Sydney, Australia)* I first met Jim Barber in 1967. I was a postdoc at the Botany School, Cambridge in the Biophysics Unit led by Dr Enid MacRobbie. And we had regular excursions to the Biophysics Department of Jack Dainty at the University of East Anglia in Norwich. Jack and Enid were the high priests of the new science of ion fluxes in plant cells using giant algal cells. Jim was doing a PhD on the ion relations of *Chlorella*, which we all thought was very brave: we had the good oil (to use an Australian slang term) and anyone who was working on those horrid small cells was to be pitied! Not me though! I chose the ion relations of the chloroplasts of giant algal cells and this brought me into direct and stimulating interaction with Jim, leading to many discussions of the ion transport and photosynthetic properties of thylakoids.

When Jim went to Leiden and then Imperial College in the late 60s I went to the Johnson Foundation, still working on the energetics of thylakoid membranes. For me it was therefore a pleasant surprise to find Jim turning from *Chlorella* to much more conventional systems—especially spinach chloroplasts, although I still feel his contribution to the ion transport relations of thylakoids was undervalued. I next went to Australia as a lecturer at Sydney University, but immediately struck up a research link with Keith Boardman and Jan Anderson at CSIRO, Canberra. They had discovered the pigment protein complexes of thylakoids, which turned out to be a very fruitful area of ideas for me and Roger Hiller at Macquarie University. By this time, I was becoming much more interested in photosynthetic electron transport than ion transport and so was Jim. So, we had much interaction; not only that but Jim forged a strong bond with several Australians (or expats), amongst whom were Jan Anderson, Fred Chow and Roger Hiller. And when Bertil Andersson came out as a postdoc with Jan in 1978, that catalysed a set of positive interactions that resonated right the way across to recent and in some cases to present times. For me one of these renewed contacts was Alison Telfer, who I had known at Oxford and had joined Jim and was doing seminal work on state transitions in chloroplasts.

Over the years our friendship blossomed into something very special and Jim became a trusted mentor. We attended many conferences and meetings together every year and got to know each other well, and our wives, Hilary and Lyn, too. At this stage Jim had become interested in the crystal structure of pigment protein complexes, which led on to his ground-breaking work on the structure of photosystem II. The work which he and I did with (now professor) Tom Bibby, on *Prochloron* (see below) was very influential and ground-breaking. Also, I well remember when Jim brought Lyn and his daughter and grandchildren out to Heron Island on the Great Barrier Reef in 2011 and we helicoptered a special fish feast from the mainland for my wife’s birthday; those were halcyon days! And, of course, we published several papers together, my favourite being the structure of a light-harvesting complex from photosystem II of the enigmatic cyanobacterium, *Prochloron*, which possesses Chl *b* as well as Chl *a* (Bibby et al. 2003b).

Sadly, Jim is no longer with us but his spirit lives on. Jim was a passionate investigator, fearless for the truth; not always therefore a comfortable co-author: a strong personality and innovative investigator who is much missed.

*Wolfgang Junge (Universität Osnabrück, Germany)* In 1971 Jim and I met at Lago Maggiore. We were strolling along different trails in the vineyards atop of Stresa. Each was muttering his talk for the next day at the Second International Congress of Photosynthesis. Highly concentrated and not aware of the landscape around us we happened to meet. Surprised to bump into someone doing the same exercise, our new acquaintanceship started with broad grins of sympathy. Walking on side by side, we discovered some things in common. We were of the same age and both had changed career. He had entered photosynthesis from chemical engineering and me from physics, and we were both chasing a then new aspect of photosynthesis, the vectorial nature of primary electron transport in the thylakoid membrane but using different spectroscopic probes. While Jim studied delayed light emission as a function of an artificially induced diffusion potential (Barber and Kraan 1970), I pioneered the use of electrochromism of intrinsic pigments as a molecular voltmeter (Junge and Witt 1968). The latter technique excelled in time resolution and versatility. It allowed to time-resolve not only transmembrane electron transfer in both photosystems but also the charge flow driving the synthesis of ATP. I continued working on electron and proton transfer related to ATP-synthesis. Jim modified and extended the luminescence-approach to investigate the role of cations on the surface potential of thylakoid membranes, affecting membrane stacking and the redistribution of photic energy between the two photosystems. It led him into a long-lasting collaboration with Bertil Andersson (Barber and Andersson 1994).

Visiting Jim’s laboratory at Imperial College in 1972 I found the corridor leading to his office lined by photographs of some dons of photosynthesis research. Jim’s ambition and sense of mission were quite obvious. With my deeply anti-establishment upbringing and for other reasons as well, our initially very good relationship cooled. Still, we met regularly at conferences. At Gordon Conferences in New England we played soccer against each other: Jim goalie of “England” and me a winger for the “Rest of the World”.

After John Walker’s first X-ray structure of the nucleotide-binding headpiece of the F_1_-ATPase (Abrahams et al. 1994), the field of proton-driven ATP-synthesis was running high. John’s structural model, although a static picture, suggested the rotary motion of the central shaft over three catalytic sites. I had previously proposed a feasible rotary mechanism for the protonic drive of its membrane complement, and we now had spectroscopic evidence in real time for active rotation of the central shaft in the headpiece (Sabbert et al. 1996). One of my students had cast our ideas in a rather simple video. In 1997 Jim and I met again at a conference in Vilm, a secluded island in the Baltic Sea and former hideaway of the central party committee of the German Democratic Republic. Jim mentioned that he urgently wanted to borrow the videotape. Suspecting the involvement of Bertil Andersson, who was then chairman of the Nobel Committee for Chemistry, I joked that I would be happy to help in such a noble cause on condition that I was invited to any forthcoming event. Jim promised, received the tape, and not long after we both happily attended the ceremony for the award of the Nobel Prize in Chemistry to John Walker, Paul Boyer and Niels Skou in Stockholm. Whether the modest videotape was helpful to the committee I do not know. But on the morning of the very event, the video was broadcast on Swedish children’s TV (“barnkontoret”).

Water oxidation is the source of aerobic life on earth. Its catalysis has been the most mysterious and thus the hottest topic of photosynthesis research. It was Horst Witt’s, my PhD supervisor’s, lifelong challenge (Junge and Rutherford 2007). After his formal retirement Horst had focused on the isolation, crystallization and structural analysis of photosystem II from a cyanobacterium (Zouni et al. 2001). Following Witt’s track, Jim joined forces with the crystallographer So Iwata, and in 2004 they presented the most comprehensive structural model of photosystem II so far determined, including details of the catalytic Mn_4_Ca cluster (Ferreira et al. 2004). Jim’s paper integrated the full complement of the then available knowledge from X-ray spectroscopy and metal–organic chemistry. His model, which was ahead of its time, stimulated a critical assessment by several other groups. They used quantum chemical methods and conventional as well as time-resolved and damage-free X-ray crystallography and spectroscopy (e.g., using a free electron laser). This burst of activity greatly advanced both the structural and kinetic understanding of dioxygen production [for a historical review see Junge (2019)].

With this one great leap forward, Jim turned into Mr. Oxygenic Photosynthesis. He lectured and wrote great articles on this fabulous invention of nature (Barber 2016, 2017). They were distinguished by a particular blend of excitement and clarity and were well received by a wide scientific audience. Despite his growing health problems Jim sustained his scientific activity with collaborations in Italy and Singapore. He continued to hold annual meetings in his lab. Both, the unpretentious style and scientific class of these meetings remained impressive. From 2012 on I enjoyed another series of meetings that he arranged in Singapore’s Nanyang Technological University where Bertil Andersson then served as president.

I will miss Jim’s strong, usually cheerful character, his style and sense of mission.

*Bertil Andersson (Nanyang Technological University, Singapore)* Professor James Barber was absolutely one of the key contributors to Photosynthesis Research for over 4 decades. He made an array of essential contributions not the least to the understanding of the structure of photosystem II. But he has also in a way personalized photosynthesis research by his unique ability to interact with people and to create arenas for scientific exchange. Jim is no longer with us, and his absence is felt by everyone in our field.

My interactions with Jim started around 40 years ago when I had just finalized my PhD degree under the supervision of Professor Per-Åke Albertsson at Lund University. I defended my thesis in 1978. Like many of the biochemists in Sweden at that time, we were developing biochemical separation methods. My project was to develop the use of aqueous polymer 2-phase partition for the separation of thylakoid membranes. We could for the first time isolate inside-out thylakoid membrane vesicles and also membrane sub-fractions highly enriched in photosystem II and others containing basically only photosystem I (PSI).

When I was preparing my PhD thesis, I boldly suggested a model where the two photosystems were largely separated from each other and confined to different regions of the thylakoid membrane system: PSII to the appressed grana stacks and PSI to the stroma exposed regions. At that time the dogma was that the two photosystems were arranged in a tight supercomplex. So, my model was controversial which in my naivety I was not entirely aware of. A few years later I refined my thesis model together with Jan Anderson in Australia, but when we presented the results at a photosynthesis conference in 1980 the advice was to throw the model into the waste bin.

But let’s go back again to 1978 and Lund University. After a successful defence of my thesis, I decided to send a copy of the thesis to some of the more established professors in photosynthesis at that time. The response was quite a disappointment—silence! Maybe they were not very impressed by my heretical thylakoid model. But after 2–3 months I actually received one letter. A real letter within an envelope with stamps of Queen Elizabeth (no e-mails back in those days). The letter was from Professor James Barber at Imperial College in London. I remember I was very excited. It was a very long letter, many tightly written pages. Jim started by apologizing that he had responded so late (not a big problem as the other professors did not answer at all). He congratulated me on my thesis which he thought was very good and innovative. He was in particular intrigued about the new thylakoid model and its lateral heterogeneity. No mention about any waste bin. The famous Professor Barber liked the model. Great!! But Jim went further in his letter. He wrote that he had similar ideas based upon theoretical considerations involving the effect of surface charges. In other words, Jim had reached similar conclusions by a completely different approach to our sub-fractional analyses. As a new PhD I was very encouraged by this letter from Jim and it definitely contributed to my decision to remain in research.

That letter started a long relationship, but it took until 1980 until we really met. In the first phase of our relationship Jim was the famous super professor but it soon evolved into a phase when we were colleagues (we both collaborated and competed) and through the years we became very close personal friends.

If one had the opportunity to interact with Jim and get to know him well, it was evident that he was genuinely passionate about science—completely dedicated. His long letter to the young Swedish Biochemist back in 1978 is just in line with that. On the other hand, many people saw him maybe more as a science manager and operator of a large research group with lots of funding. That was far from the full picture, according to my experience.

Jim was always determined to achieve his ambitious goals. In 2004 he managed with his group at Imperial College to solve the structure of PSII, the engine of life as he called it. His determination and inner drive had prevailed in a significant way.

**Fig. 9:**
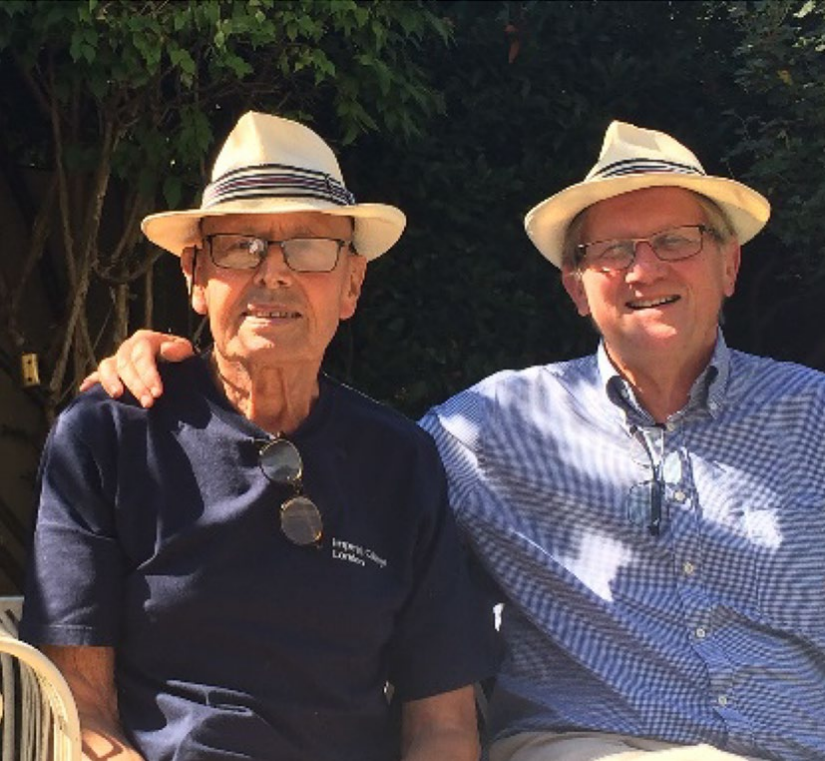
Jim and Bertil Andersson on a sunny day in London, September 2019

I am grateful that I had the privilege to know and interact with Jim for over 40 years: he was always an inspiration and it was fun. Great memories to keep (Fig. 9).

*Yasusi Yamamoto (Okayama University, Japan)* I became a postdoc of Jim in the spring of 1980. I was one of the first postdocs Jim hired after he became a full Professor at Imperial College London. In the autumn of 1979, I happened to read the advertisement for the postdoctoral position in *Nature* in the library of the Kettering Institute in Ohio, USA, where I was spending the first year as a postdoc of Bacon Ke. I sent an application letter to Jim immediately, and Jim called me soon after. In the call I could not catch what Jim said because he had a typical British accent and spoke too fast so that I was barely able to understand that he was going to accept me. That was the start of the long friendship between us. The relationship was first between a young ambitious professor and his postdoc, then between colleagues, and later between competitors, advisers and supporters. Whatever the relationship was, we understood each other very well, I believe. I was in Jim’s lab only for 13 months and studied the membrane fluidity of the thylakoid membranes using a fluorescence probe. The research was a project of the UK Agriculture and Food Research Council. I returned to Kyushu University, Japan in 1981 to start my own work to identify and to purify PsbO, PsbP and PsbQ in PSII. Jim then came to Japan twice to see me, once in Kyushu and once in Okayama. Of course, we met at many international meetings, and I was always inspired by Jim’s excellent talks and heated discussions. Thirty-five years after we first met, Jim invited me to his last gathering at Imperial College in the summer of 2015. Unfortunately, he was not in good health. I had an opportunity to give a short talk in front of Jim and many former co-workers, postdocs and students. I talked about the nice unforgettable days I spent in Jim’s lab in 1980 and the later progress of my research. After the day-long meeting, we had a small drinks party in the beautiful sunset. Jim walked over to me and we had a short conversation. “I don’t say Goodbye, Yasusi!” Jim said cheerfully. “OK, Jim. Take care!” I said. Deep sorrow came over me.

*Wah Soon (Fred) Chow (The Australian National University, Australia)* Jim Barber was at first an examiner of my thesis and then my postdoctoral supervisor. His was an active lab which I joined in 1979–1981. I worked with Jim and his lab members such as Barry Rubin and Bob Ford on experimental and theoretical approaches to understanding mechanisms underlying cation control of thylakoid stacking and the maximum chlorophyll fluorescence yield; Alison Telfer and David Chapman on state transitions in leaves; and Barry Rubin and Yasusi Yamamoto on the lateral diffusion coefficient of membrane proteins. The productive lab atmosphere helped to launch my career in photosynthesis research. I remember Jim fondly for his dynamism, his enthusiasm for photosynthesis research and his ability to get things done efficiently. Jim and his wife Lyn were extraordinarily helpful to me; indeed, I stayed with them for the first six months before my wife and son came to London. He is greatly missed.

*Werner Kühlbrandt (Max Planck Institute of Biophysics, Frankfurt, Germany)* Jim and I first met at the 6th International Photosynthesis Conference, held in 1983 in Brussels, while I was a postdoc at the ETH Zürich. Jim’s ground-breaking studies on chloroplast thylakoids and their light-harvesting complexes were the latest and most incisive contributions to a field in which he was one of the household names. Little did I think that in the years and decades to come Jim would be my mentor and one of the leading lights in my scientific life. The conference focused on reaction centres and light-harvesting complexes, and most of the pioneering researchers were there. At the time Hartmut Michel and Hans Deisenhofer were on the verge of solving the 3 Å X-ray structure of the photosynthetic reaction centre from *Rhodopseudomonas viridis* (now called *Blastochloris viridis*) (Deisenhofer et al. 1985), which would revolutionize photosynthesis research, and there was a tangible sense of excitement in the air.

For my postdoc I was investigating the structure of the plant light-harvesting complex LHC-II by electron crystallography of 2D crystals, the methods devised by my PhD mentors Richard Henderson and Nigel Unwin for purple membrane. A few months before the conference I had obtained a first low-resolution structure of LHC-II (Kühlbrandt 1984) and had also managed to grow three-dimensional crystals of the complex for X-ray crystallography. My posters at the meeting attracted a fair amount of attention from the great and the good, whom until that time I had only known from the literature. One of them was Jim Barber.

Jim’s early work at Imperial College focused on the electrochemical properties and interactions of chloroplast thylakoid membranes, which were largely determined by LHC-II as one of their main protein components. Jim had published an impressive single author paper that included a baffling array of complicated differential equations (Barber 1980b). On the basis of these theoretical studies, he developed a model of salt-induced thylakoid membrane stacking (Barber 1980a) that was much discussed and became very influential. At the same time, Jim was intent on improving experimental methods for the isolation of intact chloroplasts (Nakatani and Barber 1977).

As someone who was trying to work out the high-resolution structure of LHC-II, I had scrutinized (although, admittedly, only partly understood) Jim’s seminal treatise on thylakoid membrane electrochemistry. I was full of admiration for someone whom I imagined as an old, withered, grey-haired academic—anyone who knew that many differential equations had to be. When we met at the Brussels conference I was amazed and relieved to find that he was a tall, suntanned, youthful and totally bald figure in his early 40s. Jim was endowed with a healthy, impish sense of humour, expressed in a broad and benevolent smile, his most characteristic facial expression. I found him to be very approachable, and at the same time very sharp.

In 1984 my career at the ETH came to a sudden end, because I had dared to publish my first Nature paper on the structure of LHC-II as a single author, without the professor and formal head of our research group. I therefore had to find a new position at short notice. Richard Henderson helped me to secure an EMBO long-term fellowship, and Jim, then in the Biology Department at Imperial College London opposite the Royal Albert Hall, gracefully welcomed me as a scientific refugee in his research team. So did David Blow, the Professor of Biophysics at Imperial. Jim’s hope and expectation was that I would solve the structure of LHC-II while I was there, and he did everything he could to support me in this long-term endeavour. In the end it took another 7 years after I had left Imperial before I was able to complete the 3.4 Å cryo-EM structure of LHC-II at EMBL Heidelberg (Kühlbrandt et al. 1994). The 2.5 Å X-ray structure followed in 2005 (Standfuss et al. 2005), long after I had taken up my present position at the Max Planck Institute in Frankfurt.

Jim’s support was invaluable, and it meant a great deal to me at the outset of my research career. Amongst other things, he encouraged me to present my results and make myself known at international meetings. One memorable occasion was the 1985 Photosynthesis Gordon Conference, to which Jim had been invited, and he had asked Alison Telfer and me from his research group to come along. The three of us flew to Boston one day before the meeting, to help us get over the jet lag. Jim had hired a fairly large fancy car, but no one had thought it necessary to book accommodation for the night. From Logan airport we first headed for dinner at Legal Seafoods, his favourite place in town. We then set off in search of somewhere to stay. But every hotel was fully booked. We drove further and further out into the suburbs. Finally, when it was getting dark and we were dropping with fatigue, we chanced upon a hotel which had a lit vacancy sign outside. But the only room they had, apparently the last one in the Boston area, was a triple. We had each wanted a single room but had no option and took it. When we got to the room, our faces dropped because it only had one double and one single bed. Alison made a beeline for the single, and Jim and I looked at each other. There was nothing for it: we had to share the double. Fortunately, it was a very large king-size, and we slept like logs. In the morning, after a hearty American breakfast we set off to the conference site at Colby Sawyer College. It was a great meeting.

From my postdoc at Imperial, I progressed to a research group leader position at EMBL Heidelberg. During my 10 years in Heidelberg, I visited London frequently, not least because my wife is a Londoner, and Jim and I kept in close touch. I admired his indefatigable devotion to unravelling the structure and mechanisms of photosystem II, and he seemed to be similarly taken with my efforts to determine the structure of LHC-II. When I finally published the 3.4 Å structure, he launched a sustained effort to recruit me to the Chair of Structural Biology at Imperial College. He left no stone unturned and introduced me to a number of wealthy potential benefactors, including a well-known building magnate with whom he arranged a dinner *à trois*. The dinner, served by a butler, took place in the magnate’s fashionable St. James apartment. I do not remember much about the meal itself, but the after-dinner conversation I remember extremely well. Our host maintained that he took great interest in biophysics, and indeed practised it himself. He said he was able to make a pendulum suspended from his hand swing in a circle, either one way round or the other, by sheer concentration and willpower. Out of politeness, Jim and I kept a straight face and feigned serious interest. But for years afterwards our host’s wondrous mental energy became a running gag between us. I never found out if Jim ever obtained any funds from him. I doubt it.

**Fig. 10:**
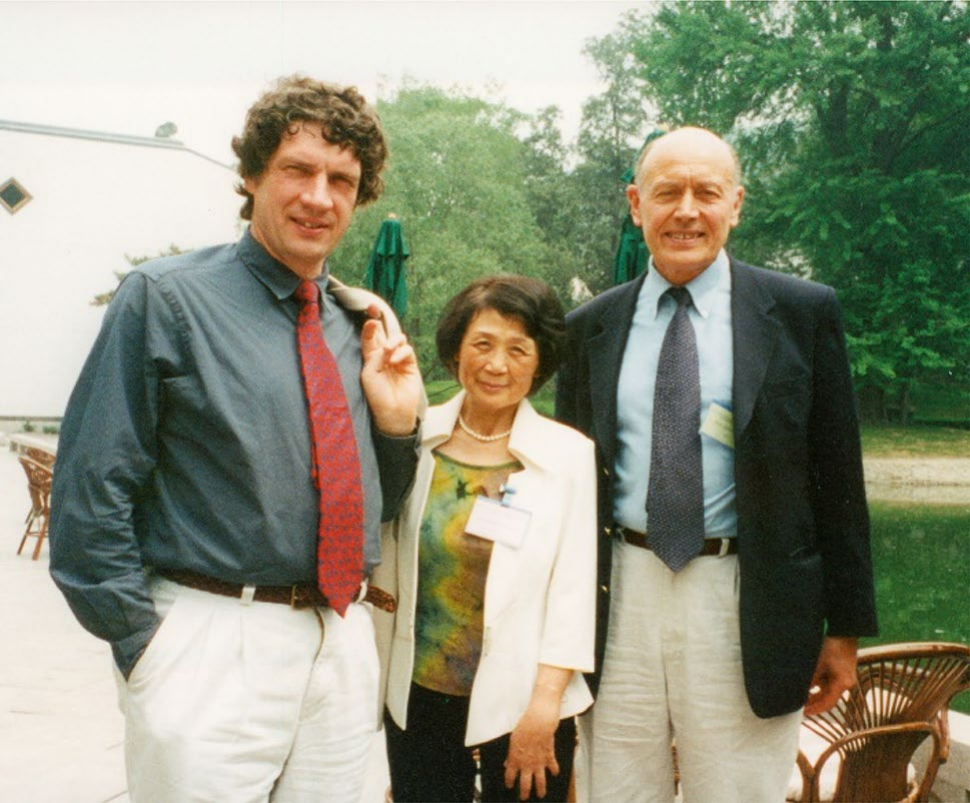
Werner, Jim and Professor Ting-Yun Kuang, Institute of Botany, Chinese Academy of Sciences at the 2010 International Photosynthesis Congress in Beijing, China

The offer of a Chair at Imperial was extremely tempting in more ways than one. In the end I had to turn it down because of a competing offer from the Max Planck Society. Jim took my decision very well. I am forever grateful to him that he did not hold it against me but remained a loyal and congenial colleague. A few years later I was in the fortunate position to support his election to the Royal Society, which I was glad to do because I knew how much it mattered to him. We continued our mutual visits to Frankfurt and London and became good friends (Fig. 10).

**Fig. 11:**
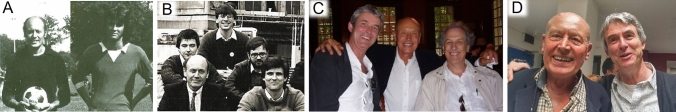
**A** With Jim (the goalie) at the 1977 Photosynthesis Congress in Reading. **B** On the steps outside the Albert Hall in about 1986, and with apologies to those who have been omitted from the original group photograph. Note the youthful Peter Nixon at the back. **C** With Jim and the late, wonderful Colin Wraight at Jim’s ‘retirement’ (he didn’t really retire) symposium, in July 2007. **D** With Jim at the Bunty meeting, Imperial College, November 2014

*Neil Hunter (University of Sheffield, UK)* I first saw Jim in 1977 at a summer school in Spetses, Greece: tanned, athletic, and radiating vitality (Jim, that is). I don’t recall talking to Jim then, but I got to know him around 1980 so we were friends for 40 years (Fig. 11). Jim’s influence on my life has been deep and lasting, so this brief tribute is more than an appreciation of his unquenchable spirit, enthusiasm and energy. I feel lucky to have known him, and luckier still that in 1984 he helped me secure my first academic position, at Imperial College. Basically, he saved me; back then, there was little sympathy in the UK for ‘old’ (that is, aged over 31 years) postdoctoral researchers who hadn’t found a university job, and you were expected to move on and find something else to do. So, when a lectureship came up at Imperial College with the potential to be affiliated with Jim’s burgeoning research group, I was quite keen (actually desperate) to be appointed; at the age of 30, I didn’t have many chances left. Jim was, as everyone knows, a man of firm opinions, and I suspect that he had exerted his considerable influence to help me secure this first position, which allowed me to embark on my career in photosynthesis research. I will always be grateful for the chance he gave me.

Jim made a great impression on me as a young academic, with his passion for photosynthesis, his utter lack of complacency, and his desire always to move forwards. Jim once told me that when he was promoted to Professor in 1980 a senior member of his department took him aside and offered some friendly advice from an old academic who had seen it all: “You’ve made it to Professor, so you can stop now”. This senior colleague clearly didn’t know Jim at all: he was just getting started and he had 600 more papers and books to write! Jim always looked to the future, never resting on his laurels; he was ambitious, restless, impatient even, and he wanted to forge ahead. No matter how much he achieved, how much praise he received, there was no self-satisfaction and only the enduring passion for his subject, photosynthesis. Even right at the end of his life Jim was consumed by finishing his book, at a time when most people would have, frankly, given up: remarkable, and typical of Jim. So, this could read merely as an account of a driven, determined man who only cared about his work, but Jim had another, and in the end more important, side to his character. This aspect of his personality earned him the affection and loyalty of his friends, and it made him loveable: Jim’s love for, and his deep pride in, his family; his real need for friendships and the ability to reciprocate and sustain them over decades; finally, Jim’s endearing self-doubt, which made this outwardly awesome, driven person human after all and the object of lasting loyalty and affection from those of us who were fortunate to know him well.

**Fig. 12:**
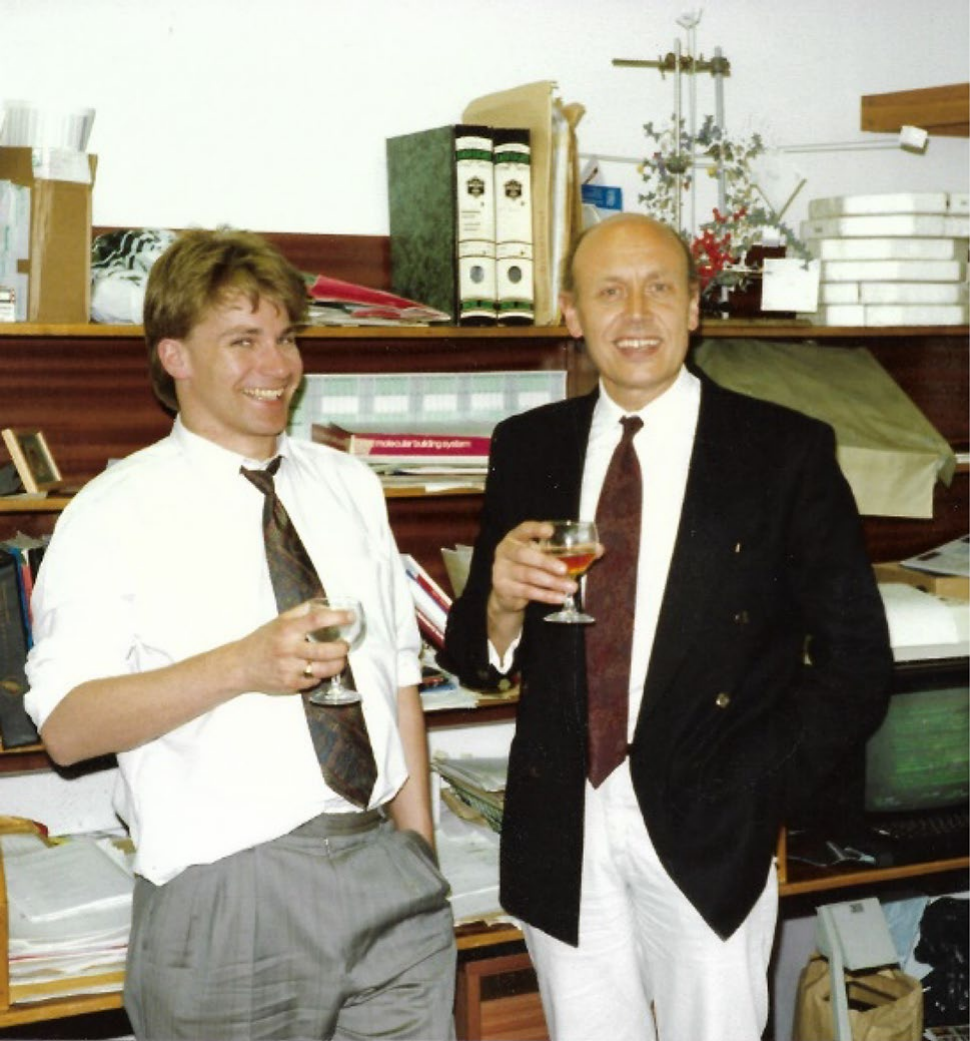
Jim and Julian Whitelegge after Julian’s successful PhD viva in 1989

*Julian Whitelegge (University of California, Los Angeles, USA)* Jim was a unique mentor who gave me a deep appreciation for the field of photosynthesis that I still embrace today. To experience his boundless energy and deep love for the subject at the start of my professional career was transformative. I was a member of his laboratory in the mid-1980s as he considered the homology between the then recently solved structure of the purple bacterial reaction center and higher plant photosystem two, and the excitement that Jim shared was remarkable (Fig. 12). I enjoyed several interactions with Jim after moving to UCLA and developing my career in membrane protein mass spectrometry, and back in London at his birthday parties. Each time I saw him I was reminded of the intense enthusiasm and passion that Jim brought to his study of photosynthesis. He was taken too young and I will miss him.

*Peter Nixon (Imperial College London, UK)* I first met Jim in the summer of 1984. All my applications for a PhD place had foundered due to lack of funding and I was desperate to find a position that would allow me to learn the emerging techniques of molecular biology as well as to work in the field of bioenergetics. Fortunately, I came across an advert Jim had placed for a PhD studentship to study photosynthetic water splitting. I was very nervous at the interview as I thought this was my last chance for a PhD place. Jim asked me a few questions about photosynthesis. Luckily, I mentioned that I had read his review on the role of surface charge in thylakoid membrane structure (Barber 1982) as it was on the reading list for my final year undergraduate course. From his reaction, I realised that this was most definitely a good thing to have mentioned. This broke the ice and I was subsequently offered the PhD position, which I gladly accepted.

It proved to be an excellent decision. Little was known about PSII at that time, so it was a period of much debate and excitement. As Jim once said: it was a time for discovering continents. My project was to study the role of the *psbD* gene product, or D2 subunit, which at the time was thought to be a quinone-binding protein, like the related D1 subunit, but with a role on the donor side of PSII. I was fortunate to have three outstanding mentors: Tristan Dyer based at the Plant Breeding Institute in Cambridge who introduced me to DNA cloning in the first year; Neil Hunter newly arrived at Imperial who invited me to continue my DNA work in his lab and Jim who with his senior postdocs (David Chapman, Alison Telfer, Paul Millner and Niki Gounaris) provided guidance on the biochemical aspects. It was a rapid learning curve and having Jim and Neil as mentors meant that I was never short of motivation and inspiration.

Jim’s lab was a magnet for overseas visitors so we were privileged to hear the latest scientific advances well before they were published in the journals. A highlight in the first year of my PhD was meeting Hartmut Michel whom Jim had invited to Imperial to give a talk on his recent determination of the structure of the bacterial reaction center (RC) (Deisenhofer et al. 1985). Towards the end of the lecture, Hartmut suggested, based on sequence similarities between the L and M subunits of the bacterial RC and the D1 subunit of PSII, that D1 was likely to be one of the two RC subunits in PSII, not just a quinone-binding subunit. But he was unclear about the identity of the second PSII RC subunit as he was unaware of the existence of the related D2 subunit. Meanwhile, Jim and I sitting in the audience were bursting with excitement after we made the connection and realised that the D2 subunit together with the D1 subunit must form the PSII reaction center. Testing this hypothesis and characterizing the properties of D1/D2 complex isolated later by Nanba and Satoh (1987) became a focus of Jim’s research for the next 10 years or so (Barber et al. 1987; Marder et al. 1987; Giorgi et al. 1996).

Besides providing an outstanding academic environment to study for a PhD, it was always fun inside and outside the lab and Jim and Lyn made sure that there were plenty of lab events, with the annual walk across Kensington Gardens just before the Christmas break for a Dim Sum lunch in Queensway a highlight. The close proximity of the Imperial College Union bar to Jim’s lab was also an extremely effective catalyst for lengthy scientific discussions amongst PhD students in the evening.

After my postdoc period working in Bruce Diner’s lab in the US, Jim encouraged me to return as a PI to Imperial College where I shared lab space with him and where I have remained to this day. Over the years, Jim became a valued colleague, mentor and friend. Jim was always encouraging, especially my research on PSII repair, which had been an area of great interest to him (Barber and Andersson 1992).

There were many characteristics that I admired about Jim. First, and foremost, was Jim’s incredible energy and drive and his determination to make a major contribution to photosynthesis research. As he once explained, he wanted to be on the stage at scientific conferences not sitting in the audience. His advice was always to focus on an important scientific problem and determining the structure of PSII was certainly that. I was always impressed by Jim’s ability to keep his research moving forwards using the latest experimental techniques, be it ultrafast spectroscopy, mutagenesis or X-ray crystallography, a lesson for all researchers.

Jim was a prolific and highly efficient writer and usually drafted papers double spaced on a lined A4 pad before passing the draft to Lyn in the next-door office to type. It seemed that it only took us 15 min to write the text of my very first paper, although making the figures was much more time consuming in those days (Nixon et al. 1986). Jim was also a great speaker who had the gift of making his research accessible and inspiring to specialists and non-specialists alike, often aided by a memorable turn of phrase such as the ‘The Big Bang of Evolution’ or ‘The Engine of Life’.

**Fig. 13:**
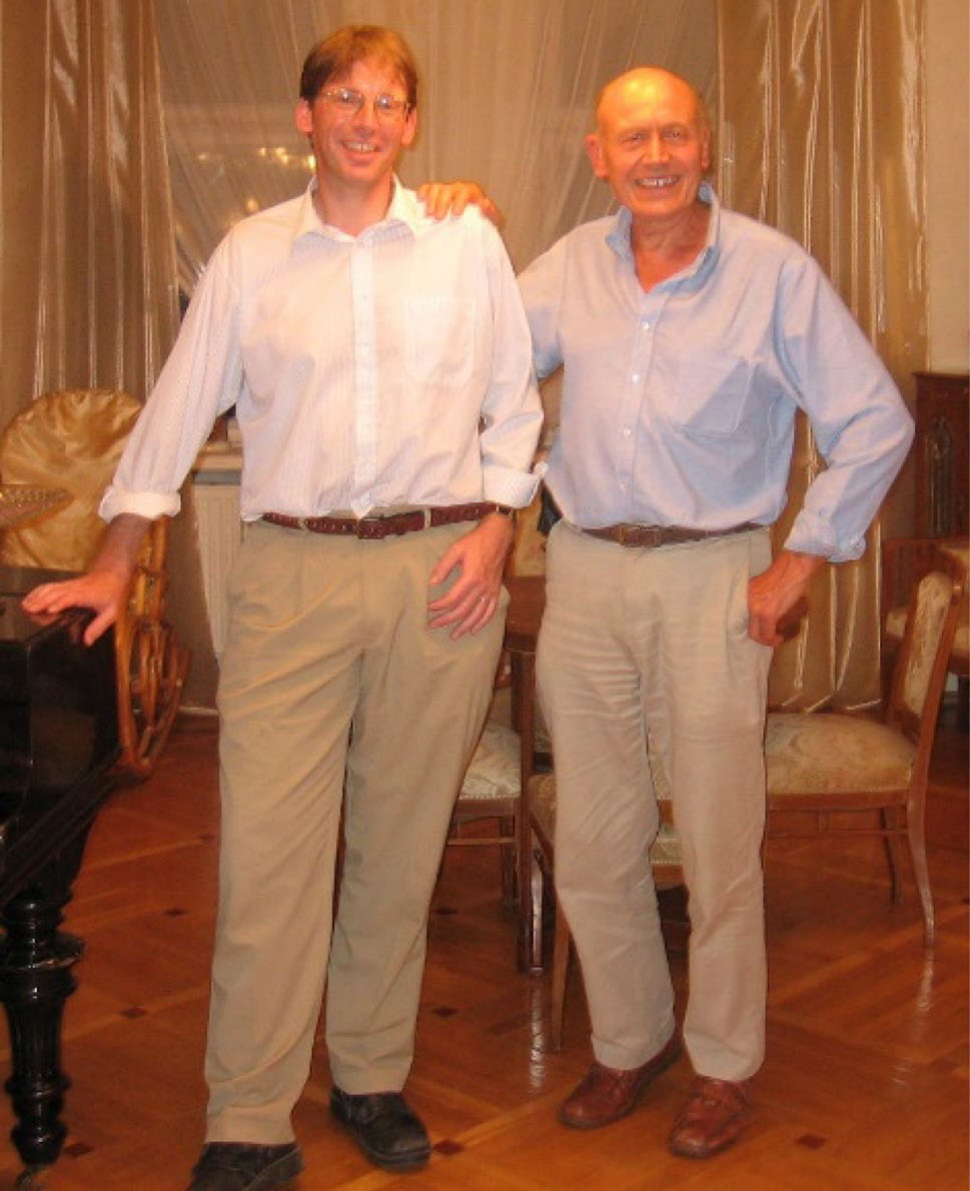
Peter Nixon and Jim in 2006 in Moscow shortly after a memorable scientific meeting in Pushchino, Russia, organised by Suleyman Allakhverdiev, Vladimir Shuvalov and the late Vyacheslav (Slava) Klimov, to mark Jim’s forthcoming retirement from Imperial College

My lasting memories of Jim will not be the science though. It will be Jim the man. It will be his indomitable spirit when he was ill. It will be the times we spent together working on his book (Barber 2020) and Jim’s reminiscences about the past, his love for his family and his great optimism that the ingenuity of humankind would lead to a brighter future (Fig. 13).

**Fig. 14:**
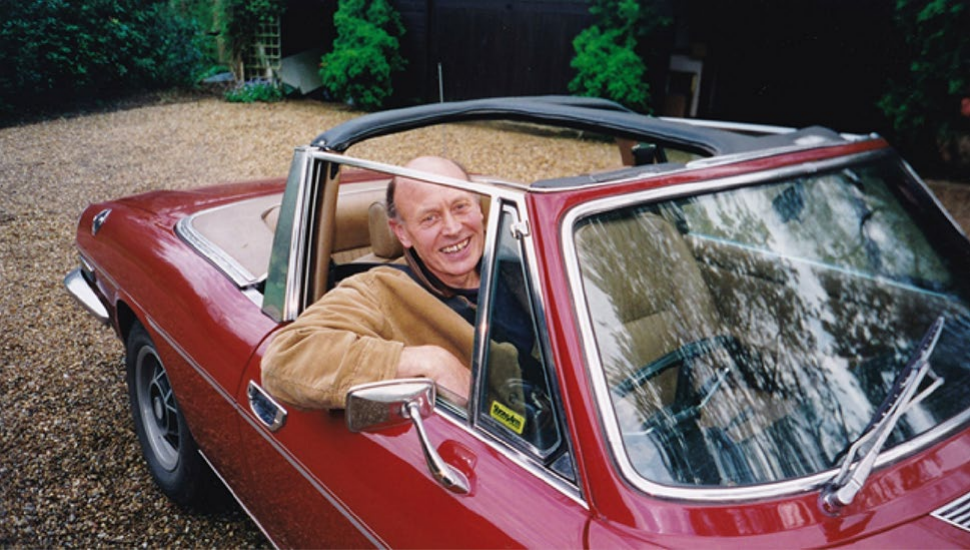
Jim about to drive his Triumph Stag, kindly taking guests back to the train station after a lab retreat at his cottage, April 1999. Photo by Jon Nield

*Jon Nield (London, UK)* It is 1991 and I am a first year biochemistry student awaiting a double lecture on Photosynthesis to be delivered by Professor Barber, Head of Department. I recall still his authoritative presentation: energetic, precise, concise. The following year I sought a research summer job, taking the lift up to his laboratories that occupied an entire seventh floor penthouse, enjoying views of distant hills and the Royal Albert Hall. Our first conversation was very relaxed. He expounded upon “The Engine of Life”, photosystem two (PSII), which apparently trumped everything, well, scientifically at least. I greatly enjoyed that summer. It led to spending a decade of being expertly mentored within Jim’s exciting, friendly and dynamic research group; gaining a Ph.D. with him on the structure of PSII; isolating and visualising photosystem supercomplexes in 3D across multiple species; countless ‘strategy’ meetings (viz. fine meals); lab writing retreats (performing live music) at his country estate (Fig. 14); jogging through Berkeley (2001; Jim was twice my age and twice as fit!); co-authoring tens of grants and papers together (1995–2016) covering so many different biochemical and biophysical techniques. If life allows for such a consistently supportive friend and mentor, then prepare to enjoy phenomenal times: thank you for all of them Jim, you are greatly missed.

**Fig. 15:**
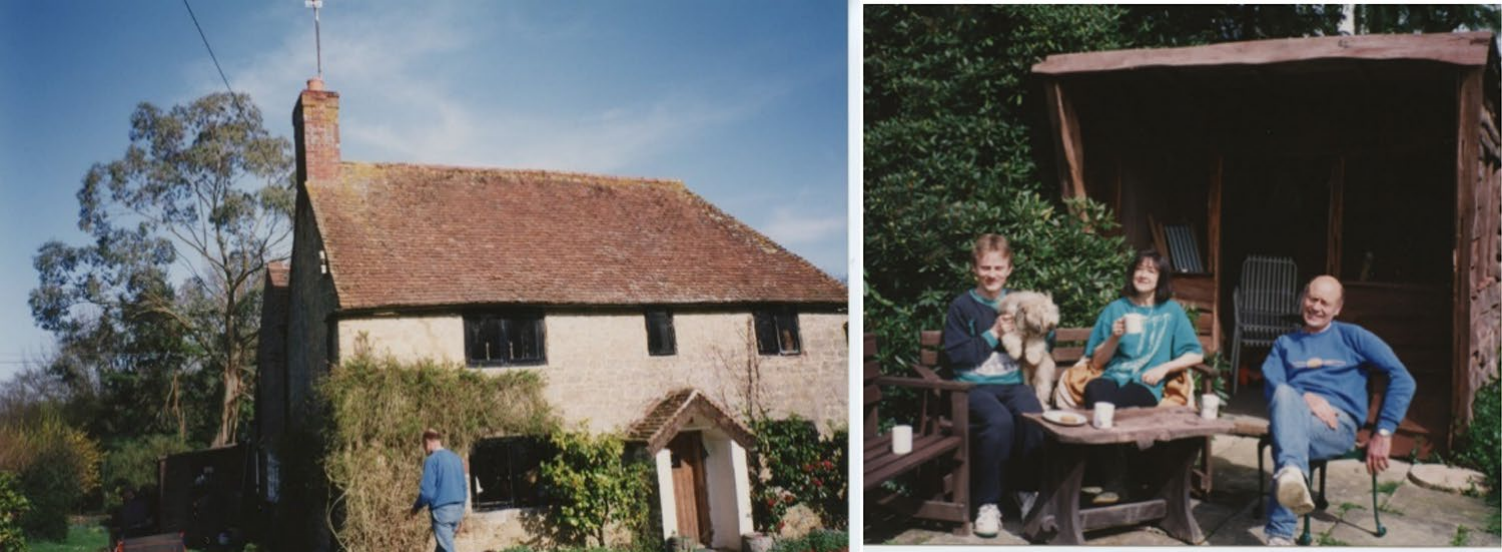
Jim at his cottage and having afternoon tea with Lyn, Josef Komenda and Eric

*Josef Komenda (Centre Algatech, Czech Republic)* I met Jim for the first time in September 1990 at the Advanced Course in Photosynthesis entitled ‘Trends in Photosynthesis Research’ in Palma de Mallorca. It was just after the discovery of the reaction centre complex of the D1 and D2 proteins as the heart of photosystem II to which Jim significantly contributed. I was a first year PhD student for the first time on a trip to Western Europe after the lifting of the Iron Curtain. Besides Jim, I met there another legend of photosynthesis research, Professor Arnon. Nevertheless, Jim immediately captured my attention by bringing the wire model of the photosystem II reaction centre with a little light, representing an electron, jumping from chlorophyll P680 via pheophytin to the quinones (Fig. 2). I was also happy that Jim found time to stop at my poster and ask me a few questions. Two years later we met again in Szeged at the Photosynthesis and Stress conference. That time Jim was looking for a postdoc fellow with experience of working on the photodamage and turnover of cyanobacterial photosystem II proteins. After a short discussion we finally agreed and in March 1993 I started my postdoctoral stay at Imperial College. At the beginning I was subjected to a strict surveillance from Jim’s side as he was apparently not sure what to expect from me. Nevertheless, when I showed him the first autoradiogram with successfully radioactively labelled proteins, he was truly excited. From that time, I obtained Jim’s full support in my research activities, and I also became a frequent guest to his cottage helping him with the gardening and playing tough pool matches with him (Fig. 15). I stayed in the lab for a year and then returned there several times for shorter stays. We also met many times at various conferences and we spent a lot of time discussing all aspects of life and science, which often inspired me in my future work. I am really grateful to Jim for giving me the chance to work with him and for introducing me to the world of international photosynthesis research. After nearly 30 years I can unequivocally state that my post-doctoral stay in Jim’s lab at Imperial College and articles published with him were crucial for the start of my scientific career and for success in my initial grant applications.

*František (Frank) Vácha (University of South Bohemia, Czech Republic)* It is truly an honor for me to share my experience of knowing such a generous, open-minded and optimistic person as Jim Barber: he will always have a special place in my heart. Most of my career, my achievements in science and beyond stem from when I first met Jim in 1991 as a Czech Ph.D. student shortly after the Velvet revolution. He offered me the opportunity of staying in his lab where I came up with a new method for isolating the PSII reaction centre complex (Vacha et al. 1995) but also, and this is probably more important, met great people, experienced good scientific practice and absorbed the way of independent life in a free country. I spent roughly a year with him in 1994/95 and visited his lab regularly for many years after that. I will also never forget visiting his country house or driving his convertible with him. Thank you, Jim, for showing me what the life of a true scientist is: I have become a professor, been a Dean of our faculty and even got a small country house and a convertible. You can be proud of me.

**Fig. 16:**
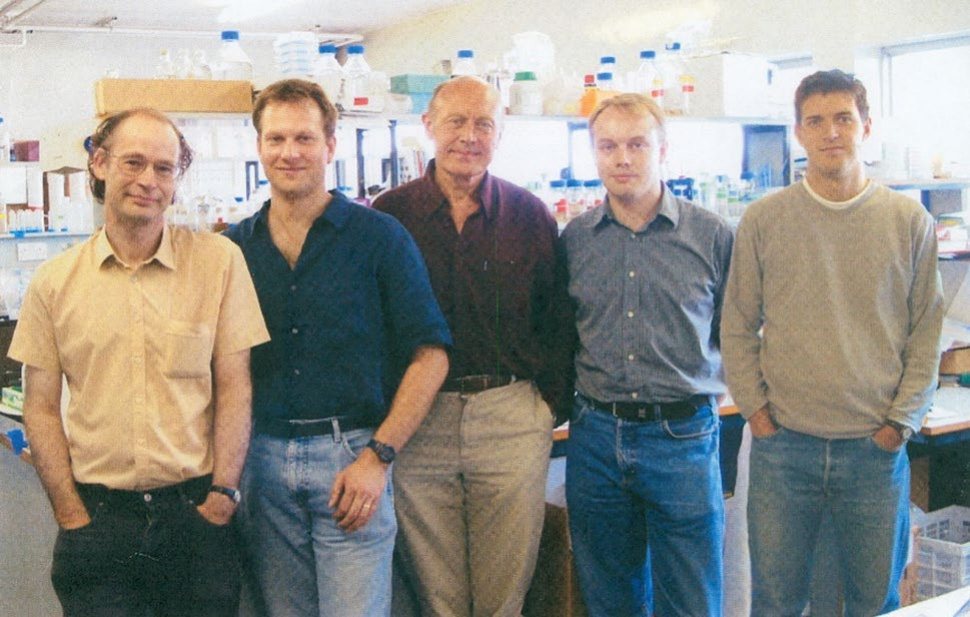
Photosynthesis Lab on the 7th floor of the Sir Ernst Chain Building in the Department of Biochemistry 2001. Left to right: Ed Morris, Ben Hankamer, Jim, Jon Nield and Tom Bibby

*Ben Hankamer (University of Queensland, Australia)* Jim was ahead of his time. He was a man of vision, thought, courage, passion and action and the deep understanding of the importance of his subject. Jim devoted his working life to defining how photosystem II had over 3 billion years, evolved to tap into the huge energy resource of the sun to drive the process of photosynthesis. Jim’s leadership was rightly recognised in many ways but despite his many achievements, the importance of his work is still not fully appreciated. For example, as world leaders meet in Glasgow for the 2021 COP26 talks to galvanize international action to tackle our climate emergency, the huge potential of photosynthesis to address this existential challenge of our time has not yet been fully grasped. This highlights just how far ahead of its time Jim’s work was, both in terms of quality and importance. The bio-inspired design of novel water splitting catalysts to drive renewable hydrogen production, new light-driven industries and the power to expand light-driven CO_2_ sequestration, are just a few of the notable examples of inspiration of his legacy to the next generations of scientists. It was a privilege to have the opportunity to be a part of the wonderful Barber team, for which I will always be immensely grateful (Fig. 16). I am sure that through the many people that Jim inspired, and I was fortunate enough to meet, the world is a better place.

*Tom Bibby (University of Southampton, UK)* I often think Jim was photosynthetic—he certainly had access to an unlimited supply of energy! I remember first meeting Jim in 1998 during my interview for a PhD position in his group at Imperial College. I recall Jim pacing his penthouse office that overlooked South Kensington and the Royal Albert Hall and explaining to me with unbounded enthusiasm and confidence how in this PhD we would solve the structure of photosystem II (PSII) and unlock the secrets of how light powers life. I was hooked both by the importance of the project but also by Jim’s unwavering can do attitude. My PhD working in Jim’s group at Imperial was a delight. I was lucky enough to join a group of hugely talented scientists all striving to understand the enigmatic process of photosynthesis. Based on the 7th floor of the Biochemistry building, to which Jim would always take the stairs two-at-a-time, it seemed that every week we would be joined by a visitor from the great and good of the photosynthesis research community—sharing knowledge, ideas and expertise. Jim had created a stimulating scientific environment that seemed to be the ‘catalytic centre’ of the photosynthetic world.

As it turns out I started my PhD with Jim not on the structure of his beloved PSII but working on the iron-stress response of cyanobacteria. Working with Jon Nield, this led to the discovery of the interaction of photosystem I (PSI) with chlorophyll-binding antenna proteins that formed an elegant functional ‘supercomplex’ and then, in collaboration with Frederic Partensky, to the discovery of similar photosynthetic supercomplexes in the globally abundant marine *Prochlorococcus*. This work with Jim, his mentorship and his continued support and advice in my early career was the foundation that has enabled me to develop my own group studying photosynthesis in the ocean.

From my time at Imperial I recall how Jim would write papers—he liked people around in the writing process, often he would drag PhD students and post-docs from the labs into in his office as he read out loud and edited drafts of the latest results. Little did I realise then that as well as honing Jim’s latest contribution to research, Jim was teaching the subject, the scientific method and process of scientific writing to the next generation of scientists. After leaving Imperial in 2002 I would regularly bump into Jim at meetings and conferences and I’d catch up on his latest impressive research. I will miss looking out for Jim in his position on the front row of these meetings, contributing vigorously to discussions of photosynthesis and striving to take the field forward.

Jim was my teacher, mentor and friend, welcoming me to his stunning cottage set in the Sussex countryside with lovely gardens (and a hot tub) first as a PhD student and more recently with my young family. Jim and Lyn would often invite the Imperial group to Sussex to escape London for a weekend. On one occasion the beer and science chat lasted into the early hours. I woke early, with a slightly sore head and stepped outside to get some air. Oddly there was a fire and smoke coming from the nearby woods. I investigated to find Jim up and active, felling timber and clearing brush—he seemed to have been up and working for some time. ‘Glad you’re up early’ said Jim with a grin, ‘grab an axe’!.

*James Murray (Imperial College London, UK)* I was doing my first postdoc when my PhD supervisor, Prof. Elspeth Garman, met Jim on a boat at Heron island, while they were both on field trips for influenza and photosynthesis, respectively. Jim and So Iwata’s groups had just published a full X-ray crystal structure of PSII, including a model of the oxygen-evolving centre (OEC), the manganese-calcium metallocluster that performs water oxidation. The electron density for the OEC showed a core with three manganese, with a bulge containing another manganese. This supported a cubane-like oxygen-evolving centre structure, as shown in Fig. 3. The cubane was substantially correct, in contrast to earlier “pair of dimers” and “butterfly” models. Later OEC models have this 3 + 1 structure, but with slightly different metal ligation. At the time there was concern about radiation damage, as metals are quickly photoreduced in the X-ray beam. My PhD was on crystallography, specialising in radiation damage. Elspeth and Jim got talking, and I later got an email from Jim, inviting me to come to talk to him.

Jim spent the entire day talking to me, and his enthusiasm for photosynthesis and PSII structure was impossible to resist. That evening by email he offered me the job, which I accepted. I made progress on PSII in Jim’s group, and he was supportive of my later projects and fellowship applications. Jim was very good at vision, leaving me to worry about the crystallographic and biochemical details, although I spent much time with Jim at the molecular graphics looking at permutations of the Mn and oxygen atoms.

Jim’s “bunty plot” meetings were a highlight of the year. He would invite world experts in photosynthesis and bioenergetics. It was fascinating to see these titans discussing and disagreeing, a happy reminder that not even Nobelists have all the answers, and that just beyond the level of the textbook, there is still much unsettled science.

In Jim’s group Joanna Kargul and Karim Maghlaoui made PSII with the calcium substituted with strontium, and anomalous diffraction showed the strontium in the same position as the calcium (Kargul et al. 2007). The original PSII structure had no chloride, although chloride and similar anions are essential for function. We made bromide substituted PSII, and again using anomalous dispersion found two bromide sites close to the OEC, which were confirmed by later higher resolution studies (Murray et al. 2008). As we now know, it is very difficult to avoid the initial steps of photoreduction in a synchrotron beam, and the problems were not meaningfully addressed until fast serial data collection with free electron lasers (XFEL) became possible. Putatively undamaged PSII structures from XFELs look very similar to synchrotron structures.

*Cristina Pagliano (Politecnico di Torino, Italy)* I met Professor James Barber for the first time at the International Congress on Photosynthesis in Glasgow in 2007, when I was fresh PhD student and he was a 67-year-old “giant” of the photosynthesis world, whom I knew only from the literature. There, I was impressed by his extremely passionate plenary lecture on PSII. On the bus to the airport, I finally had the chance to talk to him and he was very friendly. I came home enthused to have met this big professor, but I would never have believed that in the following years I would work with him and that I would also call him Jim. Fortuitously, the following year while looking for a postdoc position in the photosynthesis field, I received an offer to set up a new laboratory of photosynthesis in Italy at the Politecnico di Torino, where Jim had recently become a Visiting Professor. Unbelievably, not only had I found a postdoc on my favourite topic but, also, I had found it with him and in Italy!

Therefore, I rolled up my sleeves and planned the transformation of an ex-mechanical engineering building into a functional laboratory of plant biochemistry, under Jim’s amused gaze, who in the midst of the long months of construction works hosted me in London at Imperial College. There, he introduced me to his research project (and dream) of solving at high-resolution the structure of the “engine of life, PSII”. During that time, he also hosted me in the mansard apartment of his house in Chiswick, where I spent wonderful evenings talking to him and his lovely wife Lyn, sometimes also cooking Italian dinners for them, and occasionally for other members of the lab.

**Fig. 17:**
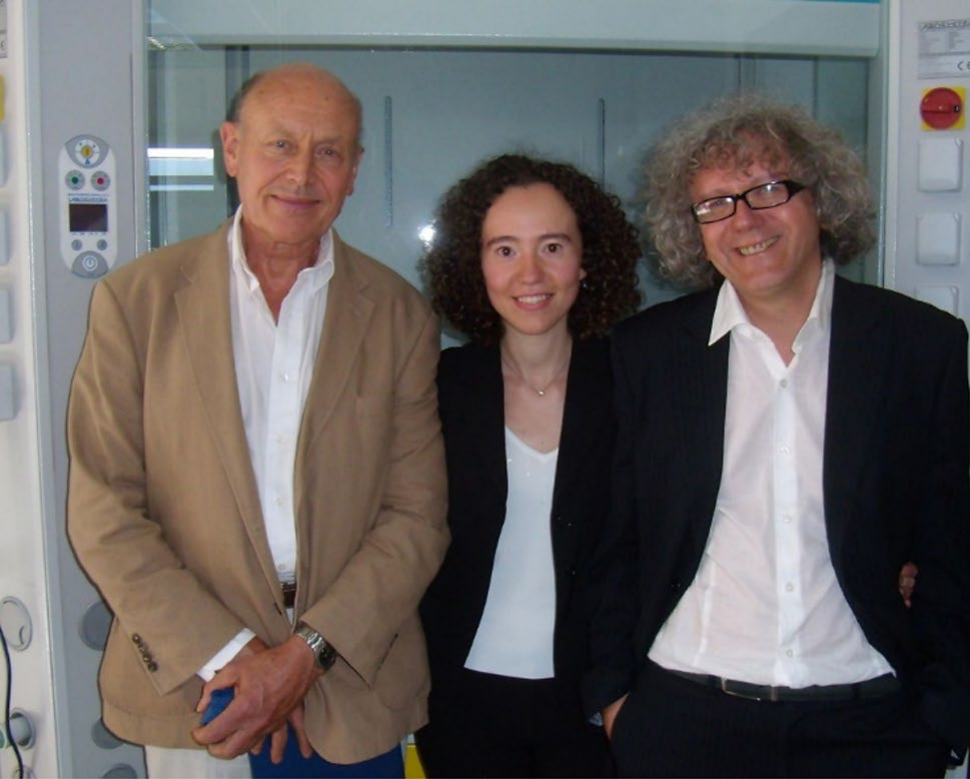
Jim, Cristina Pagliano and Roberto Barbato, who was a frequent visitor to Jim’s lab at Imperial, at the inauguration of the BioSolar Lab at the Politecnico di Torino on 4th June 2009

Once the BioSolar Lab opened (Fig. 17), I worked with him mainly from Italy, although I happily travelled to London from time to time to do experiments at Imperial College. He often came to visit the BioSolar Lab, even more frequently after buying a property on the charming Italian Orta Lake not far from the lab. Each time he was eager to be updated on the progress of the research, was proactive, full of new ideas, encouraging and always very energetic. In his office, at home or even on Lake Orta during the last meetings when he was already fighting his illness, I saw Jim always deeply immersed in writing papers and books about photosynthesis.

Thank you Jim for your extraordinary example of passion for research and tenacity in work and life. Looking at your attitude to life greatly supported my personal and professional progress and it remains the most important teaching among all your other precious tips that will bring fruits in the future.

**Fig. 18:**
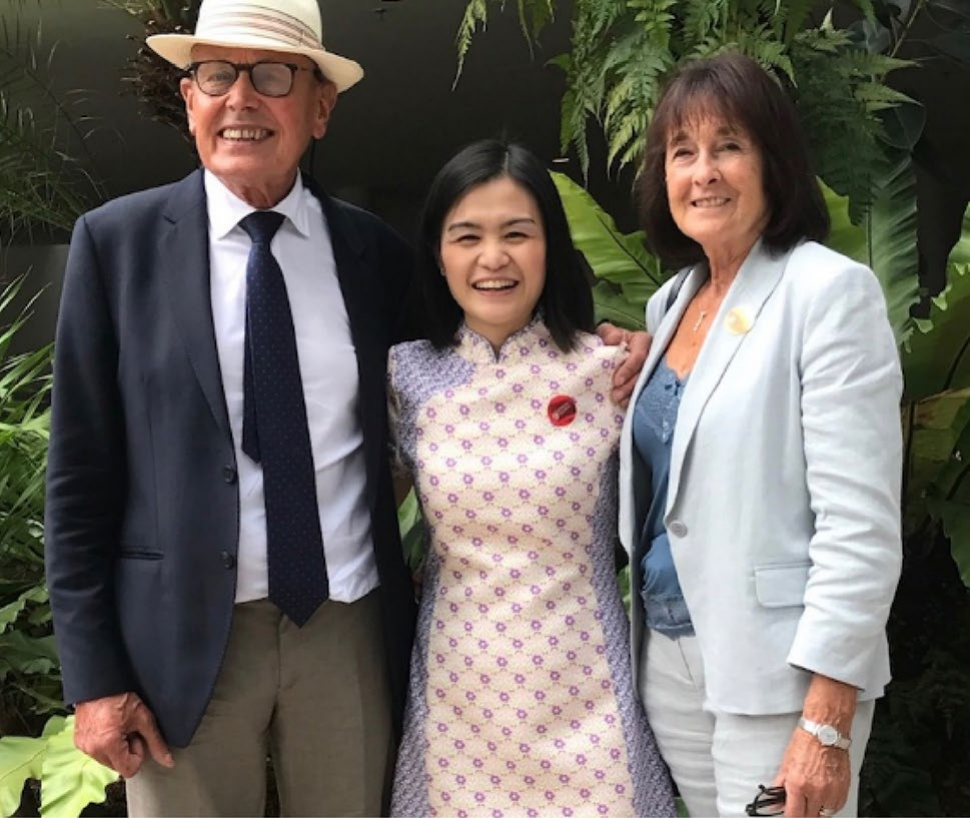
Jim, Lydia Wong and Lyn in 2017 in Singapore

**Fig. 19:**
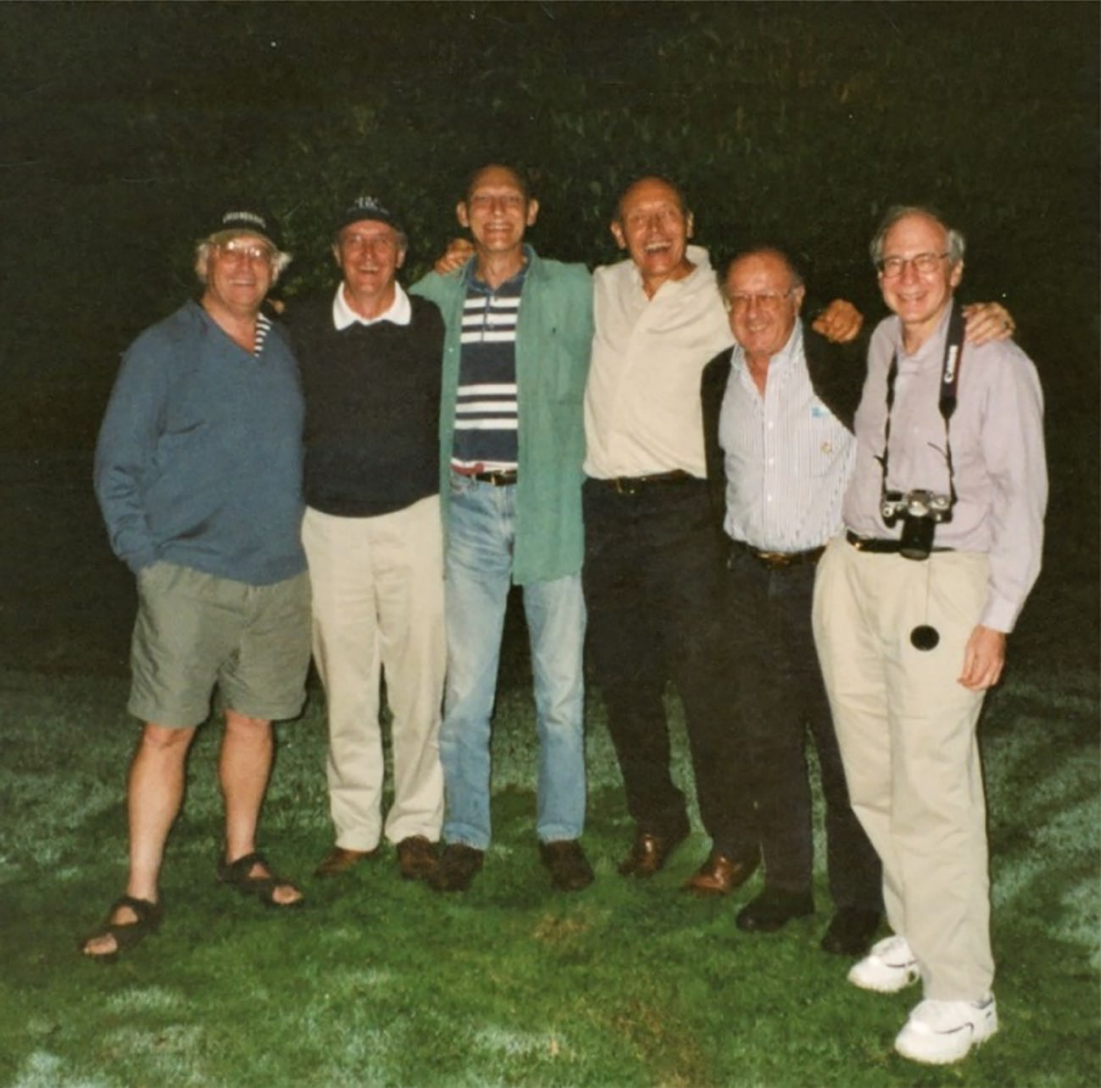
From left to right: John Walker, Les Dutton, Gerry Babcock, Jim, Janos Lanyi and Bob Gennis. Photo was taken at Jim’s cottage in 2000 during the 11th EBEC Conference at the University of Sussex. Earlier in the day, the group had visited Jim’s childhood home in Portsmouth and then proceeded to Portsmouth Historic Shipyard where they visited HMS Victory, Mary Rose and other exhibits. Thanks to John Walker for providing the anecdote

*Lydia Wong (Nanyang Technological University, Singapore)* I first met Prof Barber in 2011 when he was in Singapore as the Advisor of the newly opened Solar Fuel Laboratory at Nanyang Technological University and I was a new Assistant Professor with no experience in the field. Throughout the years, his enthusiasm and guidance have inspired me and my colleagues to discover new ways to generate cheap and clean fuels. Even after his health declined Jim continued to visit and write scientific articles with us. I last saw him in 2017 when he received his Honorary Degree of Doctor of Science from NTU (Fig. 18). Jim was a great mentor and will always have a special place in my heart and will be missed by his many friends and colleagues (Fig. 19).

## Supplementary Information

Below is the link to the electronic supplementary material.

Supplementary file1 (MP4 1175554 kb)
